# Oxidative Stress–Microbiota–Epigenetics Crosstalk: A Missing Link Between Cognition and Social Behavior in Metabolic and Neuropsychiatric Disorders

**DOI:** 10.3390/cells15010003

**Published:** 2025-12-19

**Authors:** Farzad Ashrafi, Soroor Advani, Adrián A. Pinto-Tomás, Dilip V. Jeste

**Affiliations:** 1 Functional Neurosurgery Research Center, Institute of Functional Neurosurgery, Shohada Tajrish Comprehensive Neurosurgical Center of Excellence, Shahid Beheshti University of Medical Sciences, Tehran 19899-34148, Iran; farzad.ashrafi@sbmu.ac.ir; 2 Neurology Department, Shohada Tajrish Hospital, Shahid-Beheshti University of Medical Sciences, Tehran 19899-34148, Iran; s.advani@sbmu.ac.ir; 3 Center for Research in Microscopic Structures and Biochemistry Department, School of Medicine, University of Costa Rica, San Jose 11501, Costa Rica; adrian.pinto@ucr.ac.cr; 4 Global Research Network on Social Determinants of Mental Health and Exposomics, San Diego, CA 92037, USA

**Keywords:** oxidative stress, gut microbiota, cognitive impairment, social deficits, epigenetics

## Abstract

**Highlights:**

**What are the main findings?**

- Patients with neuropsychiatric and metabolic disorders exhibit cognitive impairment or social deficits, which are associated with gut dysbiosis and aberrant gut microbiota (GM) profiles via epigenetic mechanisms.
- Gut-balancing therapies may be considered promising approaches to manage or treat cognitive impairment or social deficits by normalizing epigenetic aberrations.

**What are the implications of the main findings?**

- The bidirectional communication network linking the gut and the brain, referred to as the gut–brain–microbiota axis, plays a pivotal role in the progression of cognitive impairments and social dysfunction.
- Gut microbiota–targeted interventions—such as prebiotics, probiotics, synbiotics, postbiotics, and fecal microbiota transplantation—may ameliorate cognitive impairments and impaired social interactions by normalizing GM profiles and enhancing key epigenetically active metabolites.

**Abstract:**

Oxidative stress (OS) reflects a pathologic imbalance between excessive production of reactive oxygen species (ROS) and insufficient antioxidant defenses. Growing evidence indicates that a healthy gut microbiota (GM) is essential for regulating redox homeostasis, whereas gut dysbiosis contributes to elevated ROS levels and oxidative damage in DNA, lipids, and proteins. This redox disequilibrium initiates a cascade of cellular disturbances—including synaptic dysfunction, altered receptor activity, excitotoxicity, mitochondrial disruption, and chronic neuroinflammation—that can, in turn, impair cognitive and social functioning in metabolic and neuropsychiatric disorders via epigenetic mechanisms. In this review, we synthesize current knowledge on (1) how OS contributes to cognitive and social deficits through epigenetic dysregulation; (2) the role of disrupted one-carbon metabolism in epigenetically mediated neurological dysfunction; and (3) mechanistic links between leaky gut, OS, altered GM composition, and GM-derived epigenetic metabolites. We also highlight emerging microbiota-based therapeutic strategies capable of mitigating epigenetic abnormalities and improving cognitive and social outcomes. Understanding the OS–microbiota–epigenetic interplay may uncover new targetable pathways for therapies aimed at restoring brain and behavioral health.

## 1. Introduction

Oxidative stress (OS) is defined as an imbalance between the reactive oxygen species (ROS), reactive nitrogen species (RNS), and antioxidant levels in cells, owing to disruptions in the function of cellular organelles such as mitochondria and endoplasmic reticulum [1,2]. ROS are produced as secondary by-products of the leaky electron transport chain (ETC) at complexes I and III in mitochondria [3]. OS can contribute to the development of different disorders by inducing damage to lipids, proteins and DNA in cells [4]. Owing to its high energy requirements, the brain consumes high concentrations of oxygen and is highly sensitive to OS [5]. OS causes disruptions in the redox signaling pathway and leads to microglial dysfunction, contributing to cognitive and social deficits during neuropsychiatric disorders (NPDs) [6]. For example, in autistic mice, impaired social interaction is linked to elevated malondialdehyde (MDA) levels and reduced activities of superoxide dismutase (SOD), glutathione peroxidase (GSH-Px), and the content of GSH in hippocampus and amygdala tissues [7]. The detrimental effects of OS on the function of neuronal cells can be associated with epigenetic aberrations. For example, OS causes an imbalance between DNA methylation and demethylation and between histone acetylation and deacetylation, which is relevant to the activation of transcription factors, and, in turn, gives rise to the Alzheimer’s disease (AD)-related gene transcription in Aβ overproduction [8]. Yang et al. reported that elevated H4K12la is directly capable of activating the FOXO1 signaling pathway, increasing OS, and contributing to diabetes-induced cognitive dysfunction phenotypes [9]. The human gut microbiota (GM) is an incredibly dynamic and intricate ecological system made up of trillions of microorganisms specialized to the host [10]. This community contains bacteria, viruses, fungi, and a broad spectrum of other microbial and eukaryotic organisms. Among these, bacteria dominate, accounting for approximately 99% of all microbes in the gastrointestinal (GI) tract. Major research efforts, such as MetaHit and the Human Microbiome Project, have cataloged over 2000 microbial species present in the human GI tract [11]. The composition of the healthy GM is mainly composed of four predominant bacterial phyla, including Firmicutes, Bacteroidetes, Proteobacteria, and Actinobacteria, which together constitute almost 98% of all microorganisms [12]. This diverse microbial community plays a key role in numerous events, including the development and fine-tuning of the immune system, maintaining immune balance, orchestration of effective immune responses, defense against pathogens, promoting intestinal barrier integrity, and regulation of metabolic processes [13,14,15,16]. Ensuring a stable and resilient GM is crucial, as it improves the community’s capacity to restore balance following derangements caused by factors like unhealthy diets, excessive consumption of antibiotics or other medications, infections, and environmental stressors [17,18,19,20].

**Figure 1:**
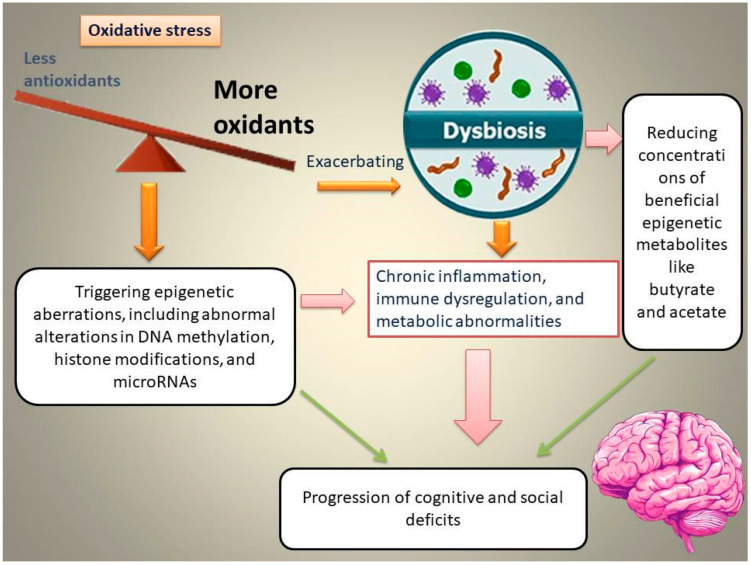
Schematic representation of the proposed pathway through which oxidative stress (OS) induces gut dysbiosis, alters epigenetic programming, and promotes the emergence and progression of cognitive and social deficits.

The intestine has attracted increasing attention from the scientific community owing to its crucial role in triggering emotional dysfunction, cognitive deficits, and neuropsychiatric conditions. Interactions between the intestine and the brain in the initiation of cognitive and social deficits are associated with OS [21]. Elevated OS in the gut is associated with gut dysbiosis and epigenetic abnormalities, which, in turn, aggravate chronic inflammation, immune and metabolic dysregulation, and subsequently, the progression of cognitive and social deficits (Figure 1).

For example, *Enterococcus* dysbiosis is linked to elevated OS in the gut, aggravating inflammatory responses, damaging the intestinal epithelium, and subsequently causing vitamin D deficiency-associated memory impairments [22]. The overgrowth of Enterococcus species is accompanied by a reduction in the levels of butyrate-producing bacteria, such as *Roseburia* spp. and *Clostridium* species, which synthesize butyrate as an epigenetic metabolite and a precursor of vitamin D3 [22]. The intestine of valproic acid (VPA)-exposed mice is underdeveloped, with excessive OS, which affects the colonization of gut microbiota (GM). It has been found that SOD supplementation during early life contributes to improved social interactions and reduced autistic behaviors by restoring autism-related GM [21]. This review examines the OS–GM–epigenetic axis as a convergent mechanism underlying cognitive and social dysfunction. We focus on (1) epigenetic consequences of OS; (2) one-carbon metabolism and homocysteine-related redox/epigenetic dysfunction; (3) interactions between leaky gut; dysbiosis; and epigenetic metabolites; and (4) microbiota-targeted therapies designed to reverse epigenetic damage and improve neurobehavioral outcomes.

## 2. Methods

Our primary objective in this review is to examine the crosstalk among OS, the GM, and epigenetic mechanisms, and to evaluate how these interactions influence cognition and social behavior in NPDs. To gather relevant evidence, we conducted independent searches in four databases, including PubMed, Web of Science, Scopus, and Embase, using the keywords “oxidative stress” or “gut microbiota” combined with “DNA methylation,” “histone modification,” or “microRNA,” along with the names of specific disorders, including cognitive impairment and social deficits. After excluding review articles and case reports, we identified more than 135 studies published from 2001 to November 2025 for further evaluation. Following a screening of the abstracts, eligible studies were examined in detail, and the extracted information is summarized in the narrative sections of this review.

## 3. Role of Oxidative Stress in Epigenetic Dysregulation and Cognitive/Social Deficits

Current research suggests that complex interactions among genetic and epigenetic factors, modulated by environmental influences, affect human vulnerability to stressors and drive the progression of cognitive and social impairments [23,24,25,26,27]. Pathological processes such as OS and inflammation can influence cognitive function and sociability through epigenetic mechanisms. In adolescents, cognitive function is shaped by the coordinated actions of epigenetic modifications, microRNA (miRNA) expression, and intracellular signaling pathways. For instance, DNA hypomethylation and elevated NF-κB activity play a critical role in determining the direction of synaptic plasticity. Moreover, the synergistic interaction of specific miRNA families, particularly miR-30 and miR-204, with the SIRT1 protein contributes to the fine-tuning of these neuroplastic processes [28]. This section discusses how OS affects cognitive/social-related genes (such as *BDNF, SIRT1, NRF2*, etc.) through specific epigenetic modifications.

### 3.1. DNA Methylation

DNA methylation is a type of epigenetic modification that affects gene expression by altering chromatin structure [29,30]. It is characterized by the addition of a methyl group (–CH_3_) to the 5-carbon position of cytosine residues, typically within CpG dinucleotides, without changing the underlying DNA sequence [30]. Pro-oxidant environmental stressors may accelerate genetic predisposition to autism and social deficits. Transmethylation defects and OS play a powerful role in autism spectrum disorder (ASD) pathogenesis [31]. For example, fine particulate matter (PM2.5) can induce pronounced redox imbalance, reduce intracellular levels of methyl donor S-adenosylmethionine, and lead to global DNA hypomethylation as well as gene-specific promoter DNA hypo- or hypermethylation, resulting in abnormal mRNA expression of autism-related candidate genes [32]. Derangements in antioxidant defenses and methylation capacity (e.g., DNA hypomethylation) have also been found in autism, which in turn increases cellular damage by influencing gene expression [33]. In ASD subjects, hypermethylation of *PGC-1α* at eight CpG sites in the promoter region of the gene, together with elevated mitochondrial DNA (mtDNA) copy number, has been associated with levels of urinary organic acids linked to mitochondrial impairment, OS, and altered neuroendocrine activity [34]. Similarly, Kulkarni et al. reported that repeated mild traumatic brain injury–induced persistent cognitive deficits may be associated with decreased mitofusin-2 (Mfn2) expression through DNA hypermethylation at the Mfn2 promoter, subsequently elevating cellular and mitochondrial ROS levels [35]. Boruch et al. identified 74 nuclear genes linked to mitochondrial glucose metabolism, fatty acid metabolism and OS pathways that exhibited altered methylation in subjects with mild cognitive impairment versus cognitively unimpaired participants. Their results showed that pronounced hypermethylation of nuclear genes involved in mitochondrial pathways may be considered a hallmark of the transition from cognitively unimpaired participants to mild cognitive impairment, whereas substantial hypomethylation at these loci may be an indicator of progression from mild cognitive impairment to AD [36]. Table 1 summarizes additional studies. In sum, OS alters DNA methylation patterns that contribute to neurobiological dysfunction and thereby develop cognitive and social impairments across neurodevelopmental and neurodegenerative conditions.

### 3.2. Histone Modifications

Histone modifications are essential epigenetic regulatory mechanisms that affect a wide range of pathophysiological processes in the human body [37]. These modifications, including methylation, acetylation, and ubiquitination, are capable of altering histones and thereby regulating gene expression [38]. In addition to DNA methylation, OS plays a powerful role in the progression of neurotoxicity and in cognitive and social deficits through changes in histone modifications [39,40]. For example, short-term OS increases the activity of Class I/II HDACs, which gives rise to a reduction in global histone acetylation [41]. Hyperhomocysteinemia-induced cognitive impairment can be caused by reduced histone H3 lysine 27 acetylation, downregulation of target genes (*Gria1*, *Gria3*, *Grin2a*, *Grin2b*, *Slc1a1*, *Slc24a2*, *Ptk2b*, and *Src*), and consequent derangements in synaptic plasticity [42].

Maternal diabetes–induced autism-like behaviors and social deficits in offspring are attributed to SOD2 suppression owing to OS-mediated histone methylation and the subsequent dissociation of early growth response 1 (Egr1) from the SOD2 promoter [43]. Liu et al. found that manganese (Mn)-induced oxidative damage and cognitive impairment in the rat striatum and in SH-SY5Y cells are linked to reduced expression of the histone acetyltransferase KAT2A and decreased H3K36ac levels in the promoter regions of antioxidant genes, including *SOD2, PRDX3,* and *TXN2* [44]. Chen et al. also found that Mn-induced neurotoxicity in rats is associated with oxidative damage in the hippocampus by reducing the expression of antioxidant genes *SOD2* and *GSTO1* via modulation of H3K18 acetylation (H3K18ac) [45]. Their findings demonstrated that increases in H3K18ac levels in the hippocampus and peripheral blood, and hence, reductions in H3K18ac enrichment at *SOD2* and *GSTO1* promoters, indicate histone acetylation changes in Mn-induced neurotoxicity. Table 1 summarizes additional studies. Overall, OS is capable of causing abnormalities in histone modifications, which in turn alter gene expression and synaptic function, and hence lead to cognitive and social impairments in neurodevelopmental and neurotoxic conditions.

### 3.3. miRNAs

MicroRNAs (miRNAs) are endogenous non-coding RNAs, approximately 21 nucleotides in length, that function as post-transcriptional regulators of gene expression [46,47]. miRNAs can either restrain or promote OS during health and disease, thereby influencing cognitive performance and social behavior [48,49]. Previous studies have shown that early exposure to OS, before and during prenatal neuronal differentiation, may contribute to the development of NPDs in adulthood through disrupting the expression of miRNAs that regulate genes critical for neurodevelopment [50]. Certain miRNAs have demonstrated the ability to target redox enzymes and hence may be excellent candidates for identifying causative factors underlying redox alterations in BD and other NPDs [51]. Owing to their capacity to interact with multiple target mRNAs within regulatory networks, small non-coding miRNAs play a pivotal role in modulating physiological processes such as behavior, OS, and neuroinflammation through gene expression regulation [49,52]. For example, Han et al. reported that cognitive impairment in diabetic rats was linked to overexpression of miRNA-23b-3p, which initiated OS, suppressed *SIRT1* and *Nrf2* expression in neurons, and reduced neuronal survival [53]. Similarly, Zhan-qiang et al. reported that miR-146a exacerbates cognitive impairment and AD-like pathology by triggering ROS production and OS via MAPK signaling [54]. In another study, Zhang et al. observed that PM2.5-exposed offspring mice exhibited elevated 8-OHG levels in microglial and Purkinje cell miRNAs at 6 weeks of age, accompanied by increased inflammatory markers, enhanced OS, and impaired cognitive performance [55]. Qu et al. demonstrated that miR-153 contributes to chronic cerebral hypoperfusion (CCH)-related cognitive impairment in male rats by negatively regulating KPNA5 expression in the basal forebrain, thereby suppressing NRF2 nuclear translocation and exacerbating OS-induced neuronal damage [56]. Moreover, participants with mild cognitive impairment exhibited elevated levels of miR-124a and miR-483-5p and decreased levels of miR-142-3p and miR-125b, which correlated with OS markers, including MDA, CAT, and SOD [57]. Table 1 summarizes additional studies. In general, miRNAs have demonstrated the ability to modulate OS and gene expression, which in turn affect cognitive function and social behavior, indicating their potential as key regulators and potential biomarkers in NPDs.

**Table 1:** Different epigenetic alterations affecting cognitive/social-related genes and pathological mechanisms.

Epigenetic Alteration	Target Genes/Key Pathological Mechanisms	Type of Study/Sample	Effects on Cognition or Sociability	Ref.
DNA methylation	*BDNF*/oxidative stress (OS)	Rats undergoing chronic unpredicted mild stress (CUMS)/brain	Homocysteine (Hcy)-induced DNA hypermethylation in the BDNF promoter reduced BDNF and caused cognitive deficits	[58]
DNA methylation	*iNOS, COX2, NFkB* and *SOD2*/neuroinflammation and OS	STZ-induced diabetic mice/the hippocampus region	Global DNA Hypermethylation was associated with diabetes-induced cognitive impairment	[59]
DNA methylation	*Syp* and *Shank2* genes	A rat model of cerebral ischemia/reperfusion injury/the hippocampus region	DNA hypomethylation enhances learning and memory recovery	[60]
Histone acetylation	Gria1, Gria3, Grin2a, Grin2b, Slc1a1, Slc24a2, Ptk2b, and Src/neuroinflammation and OS	Hyperhomocysteinemia-induced cognitive impairment model by feeding mice a high-methionine diet/the hippocampus and cortex	A considerable reduction in histone H3 lysine 27 acetylation/aberrant expression of long-term potentiation-related genes regulated by histone H3 lysine 27 acetylation is a key driver of hyperhomocysteinemia-induced cognitive impairment	[42]
Histone acetylation	*HDAC2* and *GCN5*/OS and inflammation	Social isolation stress mice/hippocampus region	Association between increased HDAC2 and GCN5 expression and social behavior dysfunction	[61]
Histone lactylation	*FOXO1* and *PGC-1α*/OS	T2DM mice and high glucose-treated microglia/brain	Increased H4K12la directly activates the FOXO1 signaling pathway, elevating OS and contributing to diabetes-related cognitive impairment	[9]
MiRNAs (miR-124a, miR-483-5p, miR-142-3p, and miR-125b)	*NO, MDA, DPP4, BDNF, SIRT-1, CAT, SOD, Bcl-2, Bax*, and *caspase-3*/OS, inflammation, and apoptosis	Healthy normal (n = 80) and mild cognitive impairment (MCI) patients (n = 70)/serum	The levels of miR-124a and miR-483-5p considerably elevated and miR-142-3p and miR-125b markedly decreased in the serum of MCI patients/The expressed miRNAs correlated positively with NO, MDA, DPP4 activity, BDNF, and SIRT-1, and negatively with the levels of CAT, SOD, Bcl-2, Bax, and caspase-3 genes	[57]
MiRNA-21	*GSK*/OS	Diabetic rats/the hippocampus region	Reduced expression of miRNA-21 was associated with derangements in brain insulin signaling and cognitive dysfunction	[62]
MiR-5699	*GRIN2B*/OS	209 unrelated patients with SCZ/blood samples	Association between disrupted MiR-5699 and cognition in patients with SCZ via OS	[63]
MiR203-5p	Glutamatergic and GABAergic genes/possibly OS	Stress-exposed C-Glud1+/− mice as a model of SCZ/medial prefrontal cortex (mPFC)	Chronic glutamate abnormalities interact with acute stress to induce cognitive deficits by increasing miR203-5p expression	[64]

## 4. One-Carbon Metabolism, Oxidative Stress, and Cognitive/Social Dysfunction

B vitamins in the one-carbon metabolism pathway (folate, vitamin B6, and vitamin B12) play an important role in the control of gene expression by modulating DNA methylation [65,66]. Their deficiency may accelerate cognitive impairment via elevated homocysteine (Hcy) levels and consequently oxidative damage [67,68]. A growing body of evidence has shown that Hcy is a potent neurotoxin and one of the main drivers of OS in the brain, causing cognitive and memory impairment by altering methylation patterns [69,70,71,72]. In the body, methionine is converted into Hcy. Increased Hcy can raise S-adenosylmethionine levels, which in turn suppress the remethylation of Hcy, leading to its further buildup [73]. This metabolic disruption initiates a cascade of OS reactions that interfere with the normal production and turnover of neurotransmitters. Vitamins B6, B12, and folate serve as key cofactors in methionine metabolism, and folate-dependent cycles play a crucial role in regulating Hcy concentrations [74]. It seems that B-vitamin deficiency and elevation of Hcy may interplay with DNA methylation of oxidative-related genes and contribute to aggravating cognitive dysfunction. For example, An et al. found that reduced serum levels of B vitamins may contribute to cognitive dysfunction by influencing methylation levels of specific redox-related genes [75]. Their findings demonstrated that there are remarkable correlations between hypermethylated sites in redox-related genes, including *NUDT15* and *TXNRD1*, and serum levels of folate, Hcy, and oxidative biomarkers [75]. Zhang et al. reported that elevation of hippocampal and serum levels of Hcy could increase mitochondrial impairment, redox imbalance, and hence cognitive damage in rats exposed to early-life stress by escalating METTL4 expression and augmenting N6-methyldeoxyadenosine (6mA) modification in mitochondrial DNA (mtDNA) [76]. In addition to DNA methylation, the detrimental effects of Hcy on cognitive function can also be mediated through alterations in histone modifications. In a study conducted by Chai et al., increased levels of the permissive histone mark trimethyl histone H3 lysine 4 (H3K4me3) and its methyltransferase KMT2B in HHcy rats were linked to disruptions of synaptic plasticity and cognitive function [77]. The authors found that H3K4 trimethylation may mediate HHcy-induced degeneration of neuronal cells by inhibiting histone acetylation through ANP32A [77]. Interestingly, increased serum Hcy levels are negatively associated with cognitive function, and this relationship may be linked to alterations in the composition of the intestinal microbial community. For example, Xu et al. reported that elevated serum levels of Hcy in patients with MDD are negatively correlated with cognitive performance and with the abundance of certain genera, including *Alistipes*, *Ruminococcae*, *Tenericides*, and *Porphyromonas* [78]. Taken together, B-vitamin deficiency and increased levels of Hcy elevate OS and epigenetic aberrations, including abnormal changes in DNA and histone modifications, which lead to cognitive impairment and neuronal dysfunction.

## 5. Interactions Between Oxidative Stress, Leaky Gut, and Gut Microbiota via Epigenetic Mechanisms

Leaky gut is a condition characterized by elevated intestinal permeability, which allows injurious microbes, endotoxins, and hazardous substances to enter the bloodstream and induce OS and inflammation in various body organs, particularly the brain [79,80]. Leaky gut and microbial changes are linked to the severity of psychological symptoms such as depression, anxiety, and cognitive and social deficits [81,82]. There is a bidirectional and cyclical association between OS and a leaky gut [83]. Excessive production of ROS disrupts the gut barrier, increasing permeability and the growth and activity of harmful bacteria. Such derangements then aggravate OS by decreasing concentrations of beneficial microbial metabolites like butyrate and elevating inflammation, which in turn accelerates the development of different diseases, especially NPDs [84]. A leaky gut disrupts gene networks in the brain relevant to immune activation, OS, and lipid oxidation—such as *Alox5*, *Lcn2*, *Mmp8*, *Nfe2l2*, *Nrros*, and *Cdkn1a*—as well as myelination in mice with colitis [85]. Qaisar et al. reported a mechanistic link between elevated intestinal permeability, higher levels of OS and inflammatory markers, and postural dysfunction, an indicator of cognitive decline, in AD subjects [86]. Their results also indicated an inverse relationship between plasma zonulin and cognitive decline in AD subjects [86]. Low sociability in alcohol-dependent patients is associated with reduced levels of butyrate-producing bacteria such as *F. prausnitzii* and elevated intestinal permeability [87]. This can be attributed to a noticeable reduction in butyrate production as an epigenetic modifier. Reduced concentrations of butyrate not only increase OS but also disrupt intestinal barrier integrity due to the critical role of this epigenetic metabolite in promoting the expression and redistribution of the tight junction proteins ZO-1 and occludin, as well as tight junction assembly [88].

In a study by Fan et al., individuals with mild cognitive impairment exhibited reduced abundance of butyrate-producing bacteria, including *Ruminococcus*, *Butyricimonas*, and *Oxalobacter*, and increased abundance of pathogenic bacteria, including Negativicutes, *Flavobacteriales*, *Gemellaceae*, and *Saccharimonadaceae*, which were associated with inflammation and OS [89]. Differential genera of GM involved in the progression of cognitive and social deficits are linked to pathological processes, particularly inflammation and OS. For example, Zhong et al. showed that ASD patients exhibited reduced mRNA levels of superoxide dismutase 2 and RAR-related orphan receptor α, elevated H3K9me2 modifications at the superoxide dismutase 2 promoter, increased amounts of 8-oxo-dG in oral epithelial cells, and a decreased reduced glutathione/oxidized glutathione (GSH/GSSG) ratio in saliva, which were associated with increased OS and altered oral microbiota [90]. More studies on the association of differential genera of GM with cognitive and social deficits and related pathological processes, especially OS, are summarized in Table 2.

## 6. Microbiota-Based Interventions Targeting Epigenetic Abnormalities

**Figure 2:**
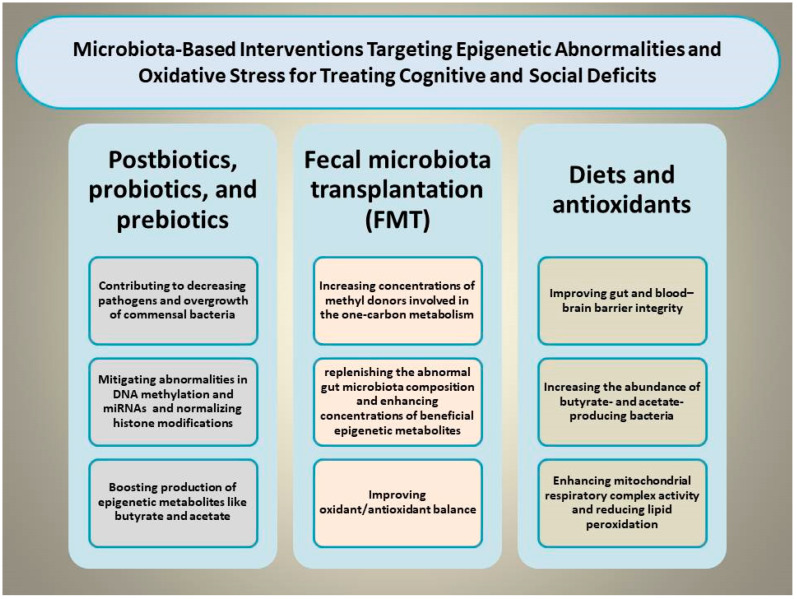
Illustration of the proposed mechanisms through which microbiota-based interventions, including prebiotics, probiotics, synbiotics, postbiotics, and fecal microbiota transplantation, may alleviate cognitive and social deficits by simultaneously normalizing epigenetic aberrations and mitigating oxidative stress.

Certain dietary and microbiota-based interventions have been found to palliate cognitive and social deficits by modulating the gut–brain axis and OS through mitigating epigenetic abnormalities [99]. For example, supplementing diverse nutraceutical compounds such as docosahexaenoic acid, vitamin D3, and probiotics exerts benefits in coping with aluminum-induced cognitive impairment by reshaping GM composition, diminishing MDA concentrations, enhancing SOD activity, and reducing glial activation [100]. This section focuses on various types of dietary and microbiota-based interventions capable of simultaneously targeting OS and epigenetic shifts to rescue cognitive and social deficits (Figure 2).

### 6.1. Postbiotics (SCFAs and Related Metabolites)

Postbiotics are metabiotics, biogenics, or epigenetic metabolites that are released by live bacteria or following bacterial lysis and provide health benefits [13,101,102]. For example, higher intake of gut microbiota-derived butyrate in people aged ≥60 years has been linked to better cognitive performance [103]. Gut microbiota-derived metabolites such as butyrate and acetate have antioxidant properties and can act as inhibitors of histone deacetylase activity [104,105,106]. In addition to altering histone modifications, they can also exert their beneficial effects by mitigating abnormalities in DNA methylation and miRNAs [107,108]. Gut microbiota-derived butyrate has been found to be a promising agent in affective disorders characterized by aberrant serotonergic activity or neuroinflammation owing to its ability to suppress OS-induced disruptions of tryptophan transport [109]. Butyrate has also been shown to improve mitochondrial function (oxidative phosphorylation and β-oxidation) during OS in cell lines from boys with autism and, therefore, may be considered a promising therapeutic agent to rescue impaired social interactions by mitigating epigenetic aberrations [110]. Moreover, the neuroprotective effects of sodium butyrate may be associated with reducing excessive ROS production via NOX2 suppression and SOD1 up-regulation through the p21/NRF2 pathway [111]. Wang et al. found that sodium butyrate could prevent Aβ25–35-induced cognitive impairments in mice by improving astroglial mitochondrial function, promoting astrocyte differentiation toward the A2 neuroprotective subtype, and enhancing the lactate shuttle between astrocytes and neurons [112]. In a study by Lu et al., the benefits of sodium butyrate in the treatment of diabetic cognitive impairments in mice were associated with improving mitochondrial damage, biogenesis, and dynamics, and up-regulating phosphorylated AMPK and PGC-1α [113]. Acetate is another epigenetic metabolite produced by gut bacteria that can exert protective effects against sleep-induced metabolic and cognitive impairments, possibly through suppressing OS [114]. Mechanistically, acetate is capable of binding and activating pyruvate carboxylase, which in turn contributes to the restoration of glycolysis and the tricarboxylic acid cycle [114]. Both acetate and butyrate promote β-cell metabolism and mitochondrial respiration under OS conditions [115].

Wen et al. found that acetate could rescue perioperative neurocognitive deficits in aged mice by suppressing the expression of inflammatory cytokines (TNF-α, IL-1β, and IL-6), OS markers (NADPH oxidase 2, inducible nitric oxide synthase, and ROS), and signaling molecules (nuclear factor kappa B and mitogen-activated protein kinase) in the hippocampus [116]. Moreover, acetate is capable of reversing social deficits by altering transcriptional regulation and improving OS in different regions of the brain [117]. Mechanistically, acetate may ameliorate impairments in social interactions by reducing histone deacetylase (*HDAC2*) gene expression, suppressing neuroinflammation by decreasing the density of Iba1+ cells and IL-1β gene expression in the hippocampus, and increasing levels of serum β-hydroxybutyrate as an antioxidant agent [118]. In conclusion, postbiotics, particularly gut microbiota-derived metabolites such as butyrate and acetate, demonstrate considerable neuroprotective and metabolic benefits, including improving antioxidant defense, epigenetic modulation, and promoting mitochondrial function.

Although postbiotics exhibit promising neuroprotective and metabolic effects, their therapeutic application may encounter several limitations. First, the high variability in the bioavailability and stability of postbiotics in vivo and poor delivery to target tissues after oral administration may diminish their effectiveness at physiologically relevant concentrations. Second, a large portion of existing findings are based on animal or in vitro studies, and human clinical evidence is still limited, making it difficult to translate their efficacy to diverse groups or identify optimal dosing. Third, the environment-dependent effects of postbiotics, shaped by individual differences in GM composition, age, metabolic health, and genetics, constrain their generalizability across populations. Moreover, high systemic levels of SCFAs could have off-target or adverse effects, like abnormal changes in immune or metabolic pathways in ways that are not yet fully understood. Ultimately, the field needs standardized approaches for postbiotic characterization, delivery, and safety evaluation to accelerate translation into clinically robust therapies.

### 6.2. Probiotics

Probiotics are live microorganisms capable of restoring beneficial bacteria and improving various diseases when consumed at appropriate concentrations [119,120]. Probiotics, including butyrate- and acetate-producing bacteria, can improve cognitive and social deficits by suppressing OS, inflammation, and apoptotic cell death via mitigating epigenetic aberrations, since butyrate and acetate act as epigenetic modifiers [119,121,122]. Wang et al. found that Lactobacillus johnsonii BS15 (L. johnsonii BS15), a lactic acid-producing probiotic, not only improved memory-related functional proteins associated with synaptic plasticity but also elevated neurotransmitter levels and mitigated restraint stress-induced OS and mitochondria-mediated apoptosis in the hippocampus by increasing mRNA levels of tight junction proteins and reducing levels of anti-inflammatory cytokines in the intestine [123]. The psychoactive effects of L. johnsonii BS15 may be attributed to regulation of epigenetics, as lactic acid can serve as a substrate for the biochemical synthesis of butyric acid, an inhibitor of histone deacetylase activity [124]. Cheng et al. demonstrated that Lactobacillus paracasei PS23 ameliorated cognitive deficits in D-galactose-induced aging mice by increasing serotonin concentrations (5-HT) and up-regulating genes involved in neuroplasticity, anti-inflammatory, and antioxidant responses, which was associated with elevated concentrations of SCFAs such as acetic acid and butyric acid in cecal contents [125]. Wu et al. reported that treatment with human Lactobacillaceae could attenuate cognitive decline in APP/PS1 mice by elevating GSH-PX activity, reducing IL-6 and MDA expression in the brain, and simultaneously increasing levels of beneficial bacteria involved in producing epigenetic metabolites while restraining harmful bacteria in the intestine [126]. *Bacillus amyloliquefaciens* SC06 improved high-fat diet-induced anxiety-like behavior and social withdrawal in male mice by inhibiting hippocampal OS, systemic inflammation, and dysbiosis, and enhancing intestinal barrier function via up-regulation of intestinal tight junctions (ZO-1 and Claudin1) [127].

In a study by Wang et al., treatment of a rat model of ASD induced by prenatal lipopolysaccharide (LPS) injection using *L. reuteri* or LGG for three weeks post-birth rescued social deficits and anxiety-like behaviors by elevating butyric acid levels, reducing propionic acid levels, improving colonic barrier integrity via up-regulation of tight junction proteins (ZO-1, Occludin, and Claudin4), mitigating HPA axis overactivation, and suppressing OS in the colon [128]. Benefits of probiotics may be associated with the regulation of miRNAs that mediate the impact of gut microbial dysbiosis on brain structure and function. As an interesting example, Chen et al. reported that daily restraint stress for 4 weeks in mice caused gut microbial dysbiosis and cognitive dysfunction, which were linked to a decrease in hippocampal miR-124 levels. Their findings demonstrated that different probiotic mixtures considerably alleviated chronic stress–induced alterations in hippocampal miR-124 levels and cognitive dysfunction [129]. In a study conducted by Mao et al., administration of probiotics increased miR-146a expression, leading to inhibition of the BTG2/Bax signaling axis, reduced neuronal apoptosis, and attenuation of OS in postoperative cognitive dysfunction mice, indicating that miR-146a-mediated suppression of BTG2/Bax contributes to the protective role of probiotic treatment against postoperative cognitive dysfunction in mice [130].

A randomized, double-blind, controlled trial by Akbari et al. demonstrated that treatment of AD patients with probiotic milk containing *Lactobacillus acidophilus*, *Lactobacillus casei*, *Bifidobacterium bifidum*, and *Lactobacillus fermentum* for 12 weeks promoted cognitive function by reducing MDA content [131].

Similarly, Tamtaji et al. showed that probiotics, including *L. acidophilus*, *B. bifidum*, and *B. longum* combined with selenium for 12 weeks, improved cognitive performance and some metabolic profiles by elevating total antioxidant capacity [132]. In a double-blind, randomized, active-controlled trial, Hsu et al. found that multi-strain probiotics, including *Bifidobacterium longum* subsp. infantis BLI-02, *B. breve* Bv-889, B. animalis subsp. lactis CP-9, *B. bifidum* VDD088, and *Lactobacillus plantarum* PL-02, administered for 12 weeks, improved cognitive function by elevating serum BDNF, decreasing IL-1β, and increasing SOD activity [133]. Collectively, probiotics, particularly SCFA-producing strains like *Lactobacillus* and *Bacillus*, may enhance cognitive performance and social behaviors by attenuating OS, inflammation, and apoptosis, often through epigenetic modulation. Animal and human studies have shown benefits like increased neurotransmitter and epigenetic metabolite levels, enhanced intestinal barrier function, and better cognitive function. Despite these promising effects, further large-scale clinical studies are needed to optimize strain selection, dosing, and treatment protocols for therapeutic use.

### 6.3. Fecal Microbiota Transplantation (FMT)

Fecal microbiota transplantation (FMT) is a medical procedure in which a small stool sample from a donor is transferred to a recipient to directly alter GM composition and induce therapeutic benefits [134,135,136,137]. Human-derived FMT alleviated social deficits in BTBR mice by normalizing OS biomarkers and modulating specific Bacteroides species and vitamin B6 metabolism, highlighting that vitamin B6, a methyl donor involved in DNA methylation, can promote social behaviors [138]. Nirmalkar et al. reported that FMT in children with autism for 10 weeks improved social interactions by increasing beneficial bacteria (Prevotella, Bifidobacterium, and sulfur-reducing Desulfovibrio) and normalizing microbial metabolic gene expression for folate biosynthesis, OS protection, and sulfur metabolism [139]. Abuaish et al. found that combined FMT and probiotic (Bifidobacteria) administration for 22 days improved social impairments and autistic features in juvenile male rats by enhancing glutathione-S-transferase levels [140]. *Prevotella histicola*, a regulator of butyrate production, transplantation mitigated cognitive impairment in vascular dementia rats by reducing MDA content, enhancing antioxidant enzyme activities (SOD and GPX), and attenuating glial cell-related inflammation via modulation of CaMKII phosphorylation in hippocampal neurons [141]. In sum, FMT exhibits promise in improving cognitive and social behaviors by restoring GM balance, attenuating OS, and enhancing antioxidant and metabolic pathways. Evidence from animal and human studies suggests it can act in synergy with probiotics and postbiotics in supporting gut–brain health. Further research is needed to optimize protocols as it presents several limitations, including variability in donor microbiota, potential risk of transmitting pathogens and infections, fluctuating long-term results, and a lack of standardized protocols for dosing, delivery, and patient selection.

### 6.4. Prebiotics

Prebiotics are known as a class of nondigestible food components, commonly specific fibers or carbohydrates, which selectively promote the growth or activity of commensal bacteria in the gut [142,143,144].

Prebiotics have been found to maintain gut and brain homeostasis by enhancing antioxidant capacity, reinforcing immune function, promoting the expression of tight junction proteins involved in intestinal barrier integrity, reshaping the GM, modulating the production of microbiota-derived epigenetic metabolites, and upregulating brain neurotrophic factors [145,146,147,148]. Mannan oligosaccharide has been shown to improve cognitive and behavioral deficits in the 5xFAD AD mouse model by increasing the abundance of Lactobacillus and decreasing Helicobacter, promoting butyrate production, and restoring brain oxidative and redox balance [149]. Gao et al. reported that treatment with Cistanche deserticola polysaccharides for two months mitigated cognitive impairment in D-galactose-treated mice by reshaping gut microbial homeostasis, suppressing OS, and reducing peripheral inflammation [150]. Chen et al. found that camellia oil intake ameliorated AlCl_3_-induced mild cognitive impairment in rats by increasing the abundance of Ruminococcaceae_UCG014, a prominent butyrate producer, which was positively correlated with antioxidant activity and reduction in OS [151]. Yang et al. demonstrated that resveratrol-loaded selenium/chitosan nano-flowers rescued glucolipid metabolism disorder-related cognitive impairment in AD mice by restoring GM balance—modulating the abundance of Enterococcus, Colidextribacter, Rikenella, Ruminococcus, Candidatus_Saccharimonas, Alloprevotella, and Lachnospiraceae_UCG-006—thereby suppressing OS, neuroinflammation, and metabolic abnormalities such as lipid deposition and insulin resistance [152]. Li et al. reported that D-galactose-induced cognitive impairment could be alleviated by acteoside, a phenylethanol glycoside from Osmanthus fragrans flowers, via rebuilding GM structure, increasing concentrations of epigenetic metabolites such as SCFAs, suppressing OS and intestinal inflammation, and restoring intestinal mucosal barrier integrity [153]. Oral administration of red cabbage anthocyanins for 12 weeks has also been shown to ameliorate age-related cognitive dysfunction in aging mice by enriching butyrate-producing bacteria, modifying the functional profile of the microbial community, decreasing MDA levels, enhancing SOD activity, and inhibiting IL-1β and IL-6 levels in serum and brain through activation of the MAPK signaling pathway [154]. In conclusion, prebiotics, such as specific fibers and plant-derived compounds, support gut–brain health by promoting beneficial bacteria, enhancing antioxidant defenses, and regulating epigenetic metabolites. Animal studies show they can improve cognitive performance and social behavior by suppressing OS and neuroinflammation and restoring gut microbial balance. Further clinical research is needed to determine their effectiveness and optimal use in humans, as prebiotics are restricted by individual variability in GM reactions, potential gastrointestinal adverse effects, and a lack of standardized dosing and long-term clinical evidence.

### 6.5. Antioxidants

Antioxidants such as glutathione, ascorbic acid, and uric acid may exert protective effects by maintaining the production of epigenetic metabolites like butyrate by the human gut and modulating OS [155]. For example, Gao et al. found that selenomethionine could improve D-galactose-induced cognitive impairment in mice by reducing OS, correcting dysbiosis through enhanced α-diversity, modulating taxonomic structure, and increasing the relative abundances of butyrate- and acetate-producing bacteria, including Akkermansia, Dorea, Acetatifactor, Atopostipes, Enteractinococcus, and Paenalcaligenes [156]. Cognitive impairment in aircraft-noise-exposed mice is associated with increased gut and blood–brain barrier permeability, elevated LPS translocation, higher levels of systemic pro-inflammatory cytokines, and increased OS indicators in the intestine, heart, and hippocampus [157]. In a study by the same group, these cognitive deficits were mitigated by astaxanthin, a carotenoid antioxidant, through suppression of inflammation and oxidative damage in intestinal, cardiac, and hippocampal tissues, as well as improvement of gut and blood–brain barrier integrity [157]. Dong et al. reported that 2-O-β-D-glucopyranosyl-L-ascorbic acid, an ascorbic acid derivative isolated from Lycium barbarum fruits, improved cognitive dysfunction and neuroinflammation induced by a high-fructose diet by increasing the abundance of butyrate- and acetate-producing bacteria, including Lactobacillus and Akkermansia, and reducing leaky gut [158]. Similarly, Li et al. demonstrated that vitamin C supplementation alleviated cognitive dysfunction in D-galactose-treated mice by mitigating hippocampal neuronal damage and enhancing anti-inflammatory and antioxidant capacity through elevation of SCFA-producing genera [159]. In another study, Chatterjee et al. showed that vitamin K2/menaquinones, a vitamin produced by gut microbiota, administered for 21 days alleviated gut dysbiosis-associated cognitive decline by increasing the abundance of bacteria involved in epigenetic metabolite production—including Lactobacillus, Bifidobacterium, Firmicutes, and Clostridium—reducing myeloperoxidase levels in the colon and brain, and abolishing OS [160]. Taken together, antioxidants such as glutathione, vitamins C and K2, and carotenoids may protect cognitive function by reducing OS and enhancing beneficial gut bacteria that produce epigenetic metabolites such as butyrate and acetate. Animal studies indicate that antioxidants enhance gut and blood–brain barrier integrity, suppress inflammation, and promote antioxidant capacity. Further clinical research is needed to confirm their effectiveness in humans.

### 6.6. B Vitamins

Adequate intake and serum levels of B vitamins (folate, vitamin B6, and vitamin B12) are linked to better cognitive and social performance by preventing neuroinflammation and OS in brain tissue, possibly via epigenetic mechanisms, particularly DNA methylation [68,161,162,163]. OS may impair vitamin B12 uptake by promoting the formation of advanced glycation end products (AGEs), which generate additional free radicals. This creates a feedback cycle in which OS and subclinical vitamin B12 deficiency reinforce each other [164]. Lower B-vitamin status plays a key role in developing cognitive impairment by increasing Hcy levels, altering DNA methylation of specific redox-related genes involved in OS, and causing subsequent oxidative damage. Moreover, folate and vitamin B12 deficiencies have been shown to exert additive effects in impairing memory function and disrupting GM in amyloid-β–infused rats [165]. Therefore, B vitamins may be considered promising candidates for improving stress, cognitive and social impairments by reshaping GM, suppressing OS, and mitigating epigenetic abnormalities [166,167,168]. Exogenous supplementation with B vitamins and folate can prevent cognitive impairment by influencing the growth of specific bacteria, enhancing the body’s antioxidant capacity, accelerating Hcy metabolism and reducing its plasma levels [169,170,171]. Mechanistically, folic acid and its reduced or methylated derivatives may provide antioxidant benefits beyond Hcy regulation by enhancing nitric oxide production, improving vascular tone, and limiting nitric oxide synthase uncoupling. The reduced forms of folate may also directly inhibit LDL oxidation [172]. Folic acid may neutralize hydroxyl and thiol radicals, providing antioxidant action and shielding lipids from peroxidative damage caused by thiyl radical attacks [173]. Generally, adequate intake and serum levels of B vitamins, including folate, B6, and B12, are linked to better cognitive and social performance by suppressing neuroinflammation, OS, and improving epigenetic abnormalities such as altered DNA methylation. Deficiencies may cause derangements in GM, elevate homocysteine levels, and exacerbate oxidative damage, creating a feedback loop that further impairs brain function. Supplementation with B vitamins is a promising strategy to improve antioxidant capacity, regulate homocysteine metabolism, and increase beneficial gut bacteria to mitigate cognitive, social, and stress-related impairments.

### 6.7. Ketogenic Diet (KD)

The ketogenic diet (KD) is a low-carbohydrate, adequate-protein, high-fat dietary regimen that promotes the production of ketone bodies, such as acetoacetate and β-hydroxybutyrate, which can act as epigenetic modifiers [174,175]. KD has been shown to inhibit OS, endoplasmic reticulum stress, inflammation, and neuronal ferroptosis, while enhancing mitochondrial respiratory complex activity, thereby exerting protective effects against cognitive deficits in neuropsychiatric diseases [176,177,178,179,180]. In a study published by Abdel-Aziz et al., eight weeks of KD feeding mitigated AD-related cognitive dysfunctions by decreasing MDA content, enhancing SOD activity, and improving neuronal survival in the hippocampus of AD model rats [181]. Similarly, Olivito et al. demonstrated that KD could rescue social and cognitive deficits, as well as repetitive behaviors, by remodeling the gut–brain axis—elevating the relative abundance of beneficial microbiota such as *Akkermansia* and *Blautia*, suppressing plasma and brain expression of TNFα, IL-1β, and IL-6, reducing lipid peroxidation, and improving SOD activity in BTBR mouse brain regions [182]. The KD may also improve ASD-related sociability by reducing systemic inflammation, correcting gut microbial dysbiosis, enhancing microbial butyrate metabolism, and modulating BDNF-associated miRNA signaling involved in brain function (reduced levels of miR-134 and miR-132 and elevated levels of miR-375) [183]. In sum, the KD contributes to the production of ketone bodies that act as epigenetic modifiers and support brain health. It improves cognitive and social outcomes by suppressing OS, inflammation, and neuronal damage and enhancing mitochondrial function and antioxidant activity. Existing challenges of the KD include its constrained food options, which make long-term adherence complicated, and its variable effectiveness across individuals. It may also contribute to causing gastrointestinal and metabolic disturbances in some individuals. Moreover, current findings about the beneficial effects of the KD are often based on short-term or preclinical studies; thereby, its long-term safety and efficacy remain unexplored.

### 6.8. Dietary Methionine Restriction

Dietary methionine restriction is a nutritional approach centered on lowering intake of the essential amino acid methionine by limiting consumption of high-methionine animal proteins and emphasizing lower-methionine plant foods [184]. Consumption of methionine-restricted diets is associated with alterations in methylation patterns, enhancing gut health, and changing the plasma metabolomic profile in rats by modulating the GM composition [185,186]. Beneficial effects of methionine-restricted diets are linked to improving intestinal barrier function, suppressing inflammatory response, OS, and enhancing concentrations of epigenetic metabolites [187,188]. As an example, Xu et al. found that dietary methionine restriction for two months ameliorated age-dependent cognitive dysfunction in mice by increasing the abundance of putative SCFA-producing bacteria, including Lachnospiraceae, Turicibacter, Roseburia, Ruminococcaceae_UCG-014, Intestinimonas, Rikenellaceae, and Tyzzerella, as well as H_2_S-producing bacteria such as Desulfovibrio in feces, which was associated with elevated concentrations of epigenetic metabolites, including acetate, propionate, and butyrate [189]. Collectively, dietary methionine restriction ameliorates age-related cognitive dysfunction and overall metabolic health by enhancing gut health, modulating the GM, improving intestinal barrier function, suppressing OS and inflammation, and increasing epigenetic metabolites.

## 7. Conclusions and Future Perspectives

Cognitive and social deficits are defined as difficulties in thinking, learning, and interacting with others that occur in various conditions, including neurodevelopmental disorders, neurodegenerative diseases, mental illnesses, metabolic disorders, brain injury, and dementia. Advances in GM research have revealed the critical role of the gut–brain axis and GM-mediated OS in the progression of cognitive and social deficits via epigenetic mechanisms. Here, we provide a detailed overview of current evidence on the key role of GM-mediated OS in the pathogenesis of cognitive and social deficits through epigenetic alterations. These findings indicate that the pathophysiology of cognitive and social deficits is closely associated with epigenetic aberrations induced by OS, and that these aberrations can, in turn, exacerbate oxidative damage. Previous studies have shown that GM-mediated OS is linked to alterations in gut permeability, microbial diversity, relative abundances of specific taxa, epigenetic markers, and GM-derived epigenetic metabolites in both patients and animal models with cognitive and social deficits. This interplay forms a bidirectional gut–brain axis, whereby OS-induced dysbiosis affects brain epigenetics and behavior, while behavioral and stress responses feedback to further alter GM and oxidative status, creating a self-reinforcing loop that links OS, epigenetic regulation, and cognitive and social outcomes.

Despite the growing interest in microbiota-targeted interventions, several limitations hinder progress. Probiotic trials have produced inconsistent and often modest effects, possibly owing to variations in strain selection, dosing, treatment duration, and lack of control of host-specific factors. FMT, while promising in preclinical investigations, faces additional barriers such as donor–recipient incompatibility, variable engraftment, poorly defined microbial-derived factors responsible for clinical effects, and concerns regarding patient safety, like the risk of pathogen transmission. Evidence supporting prebiotics, postbiotics, and dietary interventions is still largely confined to animal studies. To advance the field, future research must include well-designed mechanistic studies and rigorously conducted double-blind, randomized controlled trials. Such studies should delineate causal pathways linking GM-mediated OS, epigenetic alterations, and neurobehavioral outcomes. Progress will also require the use of larger animal models, such as dogs and monkeys, that more closely mimic human disorders, improved preservation of human biological samples, and inclusion of robust healthy control groups. Generally, although the gut–brain connection is well established, definitive evidence on the specific OS and epigenetic mechanisms underlying GM-related cognitive and social impairments remains confined, suggesting more efforts for accurate and clinically grounded research.

A practical clinical-translation roadmap for GM-based interventions begins with robust preclinical modeling to explore specific microbial strains, consortia, or diet-responsive pathways that substantially affect cognitive function and social behavior. This stage needs germ-free and humanized models, multi-omics (metabolomics, immune profiling, transcriptomics), and mechanistic authentication to clarify targetable pathways and predictive biomarkers. From there, product development must focus on good manufacturing practice, high-stringency donor assessment and processing for FMT, and standardized formulation and dosing for probiotics or live bacteria–based therapies. In parallel, developing markers/microbial signatures, epigenetic metabolites, and neuroimmune factors is crucial for patient segmentation and indicating target engagement.

The next phase should emphasize well-designed clinical trials. Early Phase I/II studies should be randomized, placebo-controlled, and powered to assess safety, tolerability, compositional changes, and preliminary cognitive or social endpoints while precisely controlling diet, drugs, and other interfering factors. If early signals are robust and reproducible, larger multicenter trials can follow up clinically substantive outcomes, pre-specified subgroups, and prolonged effectiveness. Engagement with regulators from the outset guarantees alignment on endpoints, safety screening, and quality control for live microbial therapeutics. Generally, successful translation will need to show not only short-term improvements in behavioral abnormalities but also mechanistic clarity, reproducibility, and long-term safety, setting the stage for GM-based interventions to become robust supportive therapies or disease-altering agents in neurodevelopmental and neurocognitive care.

## Figures and Tables

**Table 2:** Linking between differential genera of GM with cognitive and social deficits and related pathological processes.

Type of Deficit/Population	Relevant Pathological Processes	Microbiota Testing Method	Key Findings of Affected Gut Bacteria	Ref.
Cognitive dysfunction/mice administered to broad-spectrum antibiotics	Oxidative stress (OS)	16S rRNA Gene Amplification and Sequencing	A reduced diversity of gut microbiota and an elevation in pathogenic bacteria/reduced concentration of glutathione and elevated levels of MDA	[91]
AlCl3-induced cognitive deficits/zebrafish	Inflammation and OS	16S rDNA high-throughput sequencing	Reduced levels of Gram-positive bacteria and elevated levels of pro-inflammatory Gram-negative bacteria/increased levels of central and peripheral pro-inflammatory cytokines and reduced amounts of GSH in the brains of zebrafish	[92]
Cognitive deficits in rats with kainic acid-induced status epilepticus	Inflammation and OS	------------------	Reducing levels of SCFA-producing bacteria/the activation of glial cells, elevation of inflammatory mediators (IL-1 β, IL-6, and TNF-α), lipid peroxidation (MDA), DNA damage, and reduction in total antioxidant ability (GSH)	[93]
Cognitive impairment/60 older (ages 55–76), obese, predominately female, African American adults	Inflammation and OS	Amplicon 16S rRNA gene sequencing	Possible relationship between certain bacteria (*Methanobrevibacter* and *P. distasonis)* and cognition via oxidative pathways/possible association between *D. invisus* and cognition via systemic inflammatory pathways/positive association between cognitive score and *Akkermansia muciniphila* as an SCFAs-producing bacterium	[94]
Cognitive impairment/mice	Inflammation and OS	16S rRNA Gene Amplification and Sequencing	Positive relationship between cognitive impairment and harmful bacteria, including the ratio of *Firmicutes to Bacteroidetes*, *Helicobacter*, *Allobaculum*, *Alistipes*, *Mucispirillum*, and *Odoribacter*	[95]
Cognitive impairment/C57BL/6J (six-week-old) male mice	OS	High-throughput 16S rDNA sequencing	Correlation between Bacteroidota and Firmicutes, cognitive function, and OS/reduced Bacteroidota/Firmicutes ratio of the aging mice vs. control	[96]
Cognitive dysfunction/schizophrenia patients (N = 68) and healthy controls (N = 72)	OS	16S RNA sequencing	SOD was negatively linked to *Eubacterium*, *Collinsella*, *Lactobacillus*, *Corynebacterium*, *Bulleidia*, *Mogibacterium*, and *Succinivibrio*, but positively correlated with *Faecalibacterium*, *Ruminococcus*, and cognitive function/*Faecalibacterium* and *Turicibacter* were positively linked to cognitive function	[97]
Social deficits and spatial memory impairment/VPA-exposed mice	Inflammation and OS	16S rRNA sequencing	Decreased Bacteroidia, increased Clostridia, with reduced short-chain fatty acid (SCFA)-producing taxa (e.g., Oscillibacter)/overexpression of pro-inflammatory mediators (IL-1β, IL-6, TNF-α) and microglial hyperactivation, alongside reduced antioxidant systems (GSH, SOD)	[98]

## Data Availability

No new data were created or analyzed in this study.

## References

[1] Das T.K., Ganesh B.P. (2023). Interlink between the gut microbiota and inflammation in the context of oxidative stress in Alzheimer’s disease progression. Gut Microbes.

[2] Houldsworth A. (2024). Role of oxidative stress in neurodegenerative disorders: A review of reactive oxygen species and prevention by antioxidants. Brain Commun..

[3] Hunyadi A. (2019). The mechanism(s) of action of antioxidants: From scavenging reactive oxygen/nitrogen species to redox signaling and the generation of bioactive secondary metabolites. Med. Res. Rev..

[4] Trofin D.-M., Sardaru D.-P., Trofin D., Onu I., Tutu A., Onu A., Onită C., Galaction A.I., Matei D.V. (2025). Oxidative stress in brain function. Antioxidants.

[5] Jelinek M., Jurajda M., Duris K. (2021). Oxidative stress in the brain: Basic concepts and treatment strategies in stroke. Antioxidants.

[6] Simpson D.S., Oliver P.L. (2020). ROS generation in microglia: Understanding oxidative stress and inflammation in neurodegenerative disease. Antioxidants.

[7] Wang Y., Zhao S., Liu X., Zheng Y., Li L., Meng S. (2018). Oxytocin improves animal behaviors and ameliorates oxidative stress and inflammation in autistic mice. Biomed. Pharmacother..

[8] Gu X., Sun J., Li S., Wu X., Li L. (2013). Oxidative stress induces DNA demethylation and histone acetylation in SH-SY5Y cells: Potential epigenetic mechanisms in gene transcription in Aβ production. Neurobiol. Aging.

[9] Yang Y., Song L., Yu L., Zhang J., Zhang B. (2025). H4K12 lactylation potentiates mitochondrial oxidative stress via the Foxo1 pathway in diabetes-induced cognitive impairment. J. Adv. Res..

[10] Afzaal M., Saeed F., Shah Y.A., Hussain M., Rabail R., Socol C.T., Hassoun A., Pateiro M., Lorenzo J.M., Rusu A.V. (2022). Human gut microbiota in health and disease: Unveiling the relationship. Front. Microbiol..

[11] Hugon P., Dufour J.-C., Colson P., Fournier P.-E., Sallah K., Raoult D. (2015). A comprehensive repertoire of prokaryotic species identified in human beings. Lancet Infect. Dis..

[12] Guo X., Okpara E.S., Hu W., Yan C., Wang Y., Liang Q., Chiang J.Y., Han S. (2022). Interactive relationships between intestinal flora and bile acids. Int. J. Mol. Sci..

[13] Nohesara S., Mostafavi Abdolmaleky H., Pirani A., Thiagalingam S. (2025). Therapeutic Horizons: Gut Microbiome, Neuroinflammation, and Epigenetics in Neuropsychiatric Disorders. Cells.

[14] Paciolla C., Manganelli M., Di Chiano M., Montenegro F., Gallone A., Sallustio F., Guida G. (2025). Valeric Acid: A Gut-Derived Metabolite as a Potential Epigenetic Modulator of Neuroinflammation in the Gut–Brain Axis. Cells.

[15] Gieryńska M., Szulc-Dąbrowska L., Struzik J., Mielcarska M.B., Gregorczyk-Zboroch K.P. (2022). Integrity of the intestinal barrier: The involvement of epithelial cells and microbiota—A mutual relationship. Animals.

[16] Yu X.-Z., Yu Y., Liu Z.-Y. (2025). Crosstalk Between Intestinal Microbiota and Host Defense Peptides in Fish. Biology.

[17] De Marco P., Henriques A.C., Azevedo R., Sá S.I., Cardoso A., Fonseca B., Barbosa J., Leal S. (2021). Gut microbiome composition and metabolic status are differently affected by early exposure to unhealthy diets in a rat model. Nutrients.

[18] Singh S., Sharma P., Pal N., Kumawat M., Shubham S., Sarma D.K., Tiwari R.R., Kumar M., Nagpal R. (2022). Impact of environmental pollutants on gut microbiome and mental health via the gut–brain axis. Microorganisms.

[19] Rubas N.C., Torres A., Maunakea A.K. (2025). The gut microbiome and epigenomic reprogramming: Mechanisms, interactions, and implications for human health and disease. Int. J. Mol. Sci..

[20] Abdolmaleky H.M., Pirani A., Pettinato G. (2025). Psychosomatic Disorders, Epigenome, and Gut Microbiota. Cells.

[21] Li J., Wang H., Qing W., Liu F., Zeng N., Wu F., Shi Y., Gao X., Cheng M., Li H. (2022). Congenitally underdeveloped intestine drives autism-related gut microbiota and behavior. Brain Behav. Immun..

[22] Vinogradova E., Jarmukhanov Z., Nurgaziyev M., Kossumov A., Nurgozhina A., Mukhanbetzhanov N., Sergazy S., Chulenbayeva L., Issilbayeva A., Askarova S. (2025). Enterococcus dysbiosis as a mediator of vitamin D deficiency-associated memory impairments. Heliyon.

[23] Nohesara S., Abdolmaleky H.M., Dickerson F., Pinto-Tomas A.A., Jeste D.V., Thiagalingam S. (2025). Associations of microbiome pathophysiology with social activity and behavior are mediated by epigenetic modulations: Avenues for designing innovative therapeutic strategies. Neurosci. Biobehav. Rev..

[24] Nohesara S., Abdolmaleky H.M., Dickerson F., Pinto-Tomás A.A., Jeste D.V., Thiagalingam S. (2024). Maternal Gut Microbiome-Mediated Epigenetic Modifications in Cognitive Development and Impairments: A New Frontier for Therapeutic Innovation. Nutrients.

[25] Bellver-Sanchis A., Pallàs M., Griñán-Ferré C. (2021). The contribution of epigenetic inheritance processes on age-related cognitive decline and Alzheimer’s disease. Epigenomes.

[26] Angelopoulou E., Koros C., Hatzimanolis A., Stefanis L., Scarmeas N., Papageorgiou S.G. (2024). Exploring the genetic landscape of mild behavioral impairment as an early marker of cognitive decline: An updated review focusing on Alzheimer’s disease. Int. J. Mol. Sci..

[27] Vogrinc D., Kramberger M.G., Emeršič A., Čučnik S., Goričar K., Dolžan V. (2023). Genetic polymorphisms in oxidative stress and inflammatory pathways as potential biomarkers in Alzheimer’s disease and dementia. Antioxidants.

[28] Lee L.-C., Su M.-T., Bao L., Lee P.-L., Tutwiler S., Yeh T.-K., Chang C.-Y. (2025). MicroRNAs modulate CaMKIIα/SIRT1 signaling pathway as a biomarker of cognitive ability in adolescents. Brain Behav. Immun.-Health.

[29] Mittelstaedt N.N., Becker A.L., de Freitas D.N., Zanin R.F., Stein R.T., de Souza A.P.D. (2021). DNA methylation and immune memory response. Cells.

[30] Younesian S., Yousefi A.-M., Momeny M., Ghaffari S.H., Bashash D. (2022). The DNA methylation in neurological diseases. Cells.

[31] Gulati S., Narayan C.L., Mahesan A., Kamila G., Kapoor S., Chaturvedi P.K., Scaria V., Velpandian T., Jauhari P., Chakrabarty B. (2024). Transmethylation and Oxidative Biomarkers in Children with Autism Spectrum Disorder: A Cross Sectional Study. J. Autism Dev. Disord..

[32] Wei H., Liang F., Meng G., Nie Z., Zhou R., Cheng W., Wu X., Feng Y., Wang Y. (2016). Redox/methylation mediated abnormal DNA methylation as regulators of ambient fine particulate matter-induced neurodevelopment related impairment in human neuronal cells. Sci. Rep..

[33] Melnyk S., Fuchs G.J., Schulz E., Lopez M., Kahler S.G., Fussell J.J., Bellando J., Pavliv O., Rose S., Seidel L. (2012). Metabolic imbalance associated with methylation dysregulation and oxidative damage in children with autism. J. Autism Dev. Disord..

[34] Bam S., Buchanan E., Mahony C., O’Ryan C. (2021). DNA methylation of PGC-1α is associated with elevated mtDNA copy number and altered urinary metabolites in autism spectrum disorder. Front. Cell Dev. Biol..

[35] Kulkarni P.G., Balasubramanian N., Manjrekar R., Banerjee T., Sakharkar A. (2023). DNA methylation-mediated Mfn2 gene regulation in the brain: A role in brain trauma-induced mitochondrial dysfunction and memory deficits. Cell. Mol. Neurobiol..

[36] Boruch A.E., Madrid A., Papale L.A., Bergmann P.E., Renteria I., Faasen S., Cook D.B., Alisch R.S., Hogan K.J. (2025). Differential DNA methylation in blood in nuclear genes that encode mitochondrial proteins in mild cognitive impairment and Alzheimer’s disease. bioRxiv.

[37] De Plano L.M., Saitta A., Oddo S., Caccamo A. (2024). Epigenetic changes in Alzheimer’s disease: DNA methylation and histone modification. Cells.

[38] Mai H.A., Thomas C.M., Nge G.G., Elefant F. (2025). Modulating Cognition-Linked Histone Acetyltransferases (HATs) as a Therapeutic Strategy for Neurodegenerative Diseases: Recent Advances and Future Trends. Cells.

[39] Yang Y., Liu Y., Zhang A.-L., Tang S.-F., Ming Q., Ao C.-Y., Liu Y., Li C.-Z., Yu C., Zhao H. (2022). Curcumin protects against manganese-induced neurotoxicity in rat by regulating oxidative stress-related gene expression via H3K27 acetylation. Ecotoxicol. Environ. Saf..

[40] Ao C., Tang S., Yang Y., Liu Y., Zhao H., Ban J., Li J. (2024). Identification of histone acetylation modification sites in the striatum of subchronically manganese-exposed rats. Epigenomics.

[41] Niu Y., DesMarais T.L., Tong Z., Yao Y., Costa M. (2015). Oxidative stress alters global histone modification and DNA methylation. Free Radic. Biol. Med..

[42] Li J., Fu Y., Gu X., Xie Q., Liu Z., Cao Z., Li L., Ren J., Li Y., Yang H. (2025). Aberrant histone acetylation and dysregulated synaptic plasticity in cognitive impairment induced by a high-methionine diet. Neural Regen. Res..

[43] Wang X., Lu J., Xie W., Lu X., Liang Y., Li M., Wang Z., Huang X., Tang M., Pfaff D.W. (2019). Maternal diabetes induces autism-like behavior by hyperglycemia-mediated persistent oxidative stress and suppression of superoxide dismutase 2. Proc. Natl. Acad. Sci. USA.

[44] Liu Y., Zeng J.-M., Zhao H., Ao C.-Y., Ao L.-H., Ban J.-Q., Li J. (2024). Mechanism of KAT2A regulation of H3K36ac in manganese-induced oxidative damage to mitochondria in the nervous system and intervention by curcumin. Ecotoxicol. Environ. Saf..

[45] Chen Z., Ao C., Liu Y., Yang Y., Liu Y., Ming Q., Li C., Zhao H., Ban J., Li J. (2024). Manganese induces oxidative damage in the hippocampus by regulating the expression of oxidative stress-related genes via modulation of H3K18 acetylation. Environ. Toxicol..

[46] DeLucas M., Sánchez J., Palou A., Serra F. (2024). The impact of diet on miRNA regulation and its implications for health: A systematic review. Nutrients.

[47] Hasan H., Afzal M., Castresana J.S., Shahi M.H. (2023). A comprehensive review of miRNAs and their epigenetic effects in glioblastoma. Cells.

[48] Rashidi S.K., Kalirad A., Rafie S., Behzad E., Dezfouli M.A. (2023). The role of microRNAs in neurobiology and pathophysiology of the hippocampus. Front. Mol. Neurosci..

[49] Zhao W., Spiers J.G., Vassileff N., Khadka A., Jaehne E.J., van den Buuse M., Hill A.F. (2023). microRNA-146a modulates behavioural activity, neuroinflammation, and oxidative stress in adult mice. Mol. Cell. Neurosci..

[50] Khavari B., Barnett M.M., Mahmoudi E., Geaghan M.P., Graham A., Cairns M.J. (2024). microRNA and the Post-Transcriptional Response to Oxidative Stress during Neuronal Differentiation: Implications for Neurodevelopmental and Psychiatric Disorders. Life.

[51] Kim H.K., Tyryshkin K., Elmi N., Feilotter H., Andreazza A.C. (2018). Examining redox modulation pathways in the post-mortem frontal cortex in patients with bipolar disorder through data mining of microRNA expression datasets. J. Psychiatr. Res..

[52] Prasad K.N. (2017). Oxidative stress and pro-inflammatory cytokines may act as one of the signals for regulating microRNAs expression in Alzheimer’s disease. Mech. Ageing Dev..

[53] Han J., Liu X., Li Y., Zhang J., Yu H. (2018). Sirt1/Nrf2 signalling pathway prevents cognitive impairment in diabetic rats through anti-oxidative stress induced by miRNA-23b-3p expression. Mol. Med. Rep..

[54] Zhan-Qiang H., Hai-Hua Q., Chi Z., Miao W., Cui Z., Zi-Yin L., Jing H., Yi-Wei W. (2023). miR-146a aggravates cognitive impairment and Alzheimer disease-like pathology by triggering oxidative stress through MAPK signaling. Neurologia.

[55] Zhang J., Yang Y., Al-Ahmady Z.S., Du W., Duan J., Liao Z., Sun Q., Wei Z., Hua J. (2023). Maternal exposure to PM2. 5 induces cognitive impairment in offspring via cerebellar neuroinflammation and oxidative stress. Ecotoxicol. Environ. Saf..

[56] Qu Y., Wu Y., Xu X., Cheng W., Zhang L., Ma J., Xu W., Chen M., Cong G., Liu J. (2025). miR-153 prevents NRF2 nuclear translocation to drive hypoperfusion-related cognitive deficits by targeting KPNA5. J. Neurosci..

[57] Al-Rawaf H.A., Alghadir A.H., Gabr S.A. (2021). Molecular changes in circulating microRNAs’ expression and oxidative stress in adults with mild cognitive impairment: A biochemical and molecular study. Clin. Interv. Aging.

[58] Wang S.-D., Wang X., Zhao Y., Xue B.-H., Wang X.-T., Chen Y.-X., Zhang Z.-Q., Tian Y.-R., Xie F., Qian L.-J. (2022). Homocysteine-induced disturbances in DNA methylation contribute to development of stress-associated cognitive decline in rats. Neurosci. Bull..

[59] Fernandes V., Preeti K., Sood A., Nair K.P., Khan S., Rao B.S., Khatri D.K., Singh S.B. (2023). Neuroepigenetic changes in DNA methylation affecting diabetes-induced cognitive impairment. Cell. Mol. Neurobiol..

[60] Shi G., Feng J., Jian L.-Y., Fan X.-Y. (2023). DNA hypomethylation promotes learning and memory recovery in a rat model of cerebral ischemia/reperfusion injury. Neural Regen. Res..

[61] Asgharzadeh N., Diziche A.N., Amini-Khoei H., Yazdanpanahi N., Korrani M.S. (2025). N-acetyl cysteine through modulation of HDAC2 and GCN5 in the hippocampus mitigates behavioral disorders in the first and second generations of socially isolated mice. IBRO Neurosci. Rep..

[62] El-Sayed N.S., Elatrebi S., Said R., Ibrahim H.F., Omar E.M. (2022). Potential mechanisms underlying the association between type II diabetes mellitus and cognitive dysfunction in rats: A link between miRNA-21 and Resveratrol’s neuroprotective action. Metab. Brain Dis..

[63] Yoshikawa A., Kushima I., Miyashita M., Toriumi K., Suzuki K., Horiuchi Y., Kawaji H., Takizawa S., Ozaki N., Itokawa M. (2021). Dysregulation of post-transcriptional modification by copy number variable microRNAs in schizophrenia with enhanced glycation stress. Transl. Psychiatry.

[64] Asraf K., Zaidan H., Natoor B., Gaisler-Salomon I. (2023). Synergistic, long-term effects of glutamate dehydrogenase 1 deficiency and mild stress on cognitive function and mPFC gene and miRNA expression. Transl. Psychiatry.

[65] Lionaki E., Ploumi C., Tavernarakis N. (2022). One-carbon metabolism: Pulling the strings behind aging and neurodegeneration. Cells.

[66] Lyon P., Strippoli V., Fang B., Cimmino L. (2020). B vitamins and one-carbon metabolism: Implications in human health and disease. Nutrients.

[67] Jiang X., Guo Y., Cui L., Huang L., Guo Q., Huang G. (2023). Study of diet habits and cognitive function in the Chinese middle-aged and elderly population: The association between folic acid, B vitamins, vitamin D, coenzyme Q10 supplementation and cognitive ability. Nutrients.

[68] Ueno A., Hamano T., Enomoto S., Shirafuji N., Nagata M., Kimura H., Ikawa M., Yamamura O., Yamanaka D., Ito T. (2022). Influences of vitamin B12 supplementation on cognition and homocysteine in patients with vitamin B12 deficiency and cognitive impairment. Nutrients.

[69] Cankurtaran M., Yesil Y., Kuyumcu M.E., Oztürk Z.A., Yavuz B.B., Halil M., Ulger Z., Cankurtaran E.S., Arıoğul S. (2013). Altered levels of homocysteine and serum natural antioxidants links oxidative damage to Alzheimer’s disease. J. Alzheimer’s Dis..

[70] Tchantchou F., Goodfellow M., Li F., Ramsue L., Miller C., Puche A., Fiskum G. (2021). Hyperhomocysteinemia-induced oxidative stress exacerbates cortical traumatic brain injury outcomes in rats. Cell. Mol. Neurobiol..

[71] Mostafa M.D., ElKomy M.A., Othman A.I., Amer M.E., El-Missiry M.A. (2022). Epigallocatechin-3-gallate enhances cognitive and memory performance and protects against brain injury in methionine-induced hyperhomocysteinemia through Interdependent molecular pathways. Neurotox. Res..

[72] Wang D., Chen Y.-M., Ruan M.-H., Zhou A.-H., Qian Y., Chen C. (2016). Homocysteine inhibits neural stem cells survival by inducing DNA interstrand cross-links via oxidative stress. Neurosci. Lett..

[73] Hermann A., Sitdikova G. (2021). Homocysteine: Biochemistry, molecular biology and role in disease. Biomolecules.

[74] Zaric B.L., Obradovic M., Bajic V., Haidara M.A., Jovanovic M., Isenovic E.R. (2019). Homocysteine and hyperhomocysteinaemia. Curr. Med. Chem..

[75] An Y., Feng L., Zhang X., Wang Y., Wang Y., Tao L., Qin Z., Xiao R. (2019). Dietary intakes and biomarker patterns of folate, vitamin B6, and vitamin B12 can be associated with cognitive impairment by hypermethylation of redox-related genes NUDT15 and TXNRD1. Clin. Epigenet..

[76] Zhang L., Xie F., Wang X., Sun Z., Wu Y., Sun Z., Zhang S., Chen X., Zhao Y., Qian L. (2025). Homocysteine induced N6-methyldeoxyadenosine modification perturbation elicits mitochondria dysfunction contributes to the impairment of learning and memory ability caused by early life stress in rats. Redox Biol..

[77] Chai G.-S., Gong J., Mao Y.-M., Wu J.-J., Bi S.-G., Wang F., Zhang Y.-Q., Shen M.-T., Lei Z.-Y., Nie Y.-J. (2024). H3K4 trimethylation mediate hyperhomocysteinemia induced neurodegeneration via suppressing histone acetylation by ANP32A. Mol. Neurobiol..

[78] Xu C.-C., Zhao W.-X., Sheng Y., Yun Y.-J., Ma T., Fan N., Song J.-Q., Wang J., Zhang Q. (2025). Serum homocysteine showed potential association with cognition and abnormal gut microbiome in major depressive disorder. World J. Psychiatry.

[79] Li F., Ke H., Wang S., Mao W., Fu C., Chen X., Fu Q., Qin X., Huang Y., Li B. (2023). Leaky gut plays a critical role in the pathophysiology of autism in mice by activating the lipopolysaccharide-mediated toll-like receptor 4–myeloid differentiation factor 88–nuclear factor kappa B signaling pathway. Neurosci. Bull..

[80] Usuda H., Okamoto T., Wada K. (2021). Leaky gut: Effect of dietary fiber and fats on microbiome and intestinal barrier. Int. J. Mol. Sci..

[81] Morena D., Lippi M., Scopetti M., Turillazzi E., Fineschi V. (2025). Leaky gut biomarkers as predictors of depression and suicidal risk: A systematic review and meta-analysis. Diagnostics.

[82] Rudzki L., Maes M. (2021). From “Leaky Gut” to Impaired Glia-Neuron Communication in Depression. Major Depressive Disorder: Rethinking and Understanding Recent Discoveries.

[83] Semenova N., Garashchenko N., Kolesnikov S., Darenskaya M., Kolesnikova L. (2024). Gut microbiome interactions with oxidative stress: Mechanisms and consequences for health. Pathophysiology.

[84] Ikeda Y., Saigo N., Nagasaki Y. (2023). Direct evidence for the involvement of intestinal reactive oxygen species in the progress of depression via the gut-brain axis. Biomaterials.

[85] Boles J.S., Krueger M.E., Jernigan J.E., Cole C.L., Neighbarger N.K., Huarte O.U., Tansey M.G. (2024). A leaky gut dysregulates gene networks in the brain associated with immune activation, oxidative stress, and myelination in a mouse model of colitis. Brain Behav. Immun..

[86] Qaisar R., Karim A., Iqbal M.S., Ahmad F., Shaikh A., Kamli H., Khamjan N.A. (2023). A leaky gut contributes to postural dysfunction in patients with Alzheimer’s disease. Heliyon.

[87] Leclercq S., Le Roy T., Furgiuele S., Coste V., Bindels L.B., Leyrolle Q., Neyrinck A.M., Quoilin C., Amadieu C., Petit G. (2020). Gut microbiota-induced changes in β-hydroxybutyrate metabolism are linked to altered sociability and depression in alcohol use disorder. Cell Rep..

[88] Peng L., Li Z.-R., Green R.S., Holzmanr I.R., Lin J. (2009). Butyrate enhances the intestinal barrier by facilitating tight junction assembly via activation of AMP-activated protein kinase in Caco-2 cell monolayers. J. Nutr..

[89] Fan K.-C., Lin C.-C., Liu Y.-C., Chao Y.-P., Lai Y.-J., Chiu Y.-L., Chuang Y.-F. (2023). Altered gut microbiota in older adults with mild cognitive impairment: A case-control study. Front. Aging Neurosci..

[90] Zhong L., Ren P., Wang H., Fu C., Feng D., Wang M., Zeng L., Yao P., Wang T. (2025). Potential association between altered oral microbiota and oxidative stress in individuals with autism. Autism.

[91] Arslanova A., Tarasova A., Alexandrova A., Novoselova V., Shaidullov I., Khusnutdinova D., Grigoryeva T., Yarullina D., Yakovleva O., Sitdikova G. (2021). Protective effects of probiotics on cognitive and motor functions, anxiety level, visceral sensitivity, oxidative stress and microbiota in mice with antibiotic-induced dysbiosis. Life.

[92] Nie Y., Yang J., Zhou L., Yang Z., Liang J., Liu Y., Ma X., Qian Z., Hong P., Kalueff A.V. (2022). Marine fungal metabolite butyrolactone I prevents cognitive deficits by relieving inflammation and intestinal microbiota imbalance on aluminum trichloride-injured zebrafish. J. Neuroinflamm..

[93] Wang X., Yang C., Yang L., Zhang Y. (2022). Modulating the gut microbiota ameliorates spontaneous seizures and cognitive deficits in rats with kainic acid-induced status epilepticus by inhibiting inflammation and oxidative stress. Front. Nutr..

[94] McLeod A., Bernabe B.P., Xia Y., Sanchez-Flack J., Lamar M., Schiffer L., Castellanos K., Fantuzzi G., Maki P., Fitzgibbon M. (2023). Comparing the gut microbiome of obese, African American, older adults with and without mild cognitive impairment. PLoS ONE.

[95] Li T., Lin L., Li C., Zheng J., Chen B., Shen Y., Ren D. (2023). Amelioration of walnut-derived novel peptides against D-galactose-induced cognitive impairment by modulating the gut microbiota composition. Food Funct..

[96] Liu Z., Fayyaz S., Zhao D., Yi Z., Huang J.-H., Zhou R.-R., Xie J., Liu P.-A., He W., Zhang S.-H. (2023). Polygonatum sibiricum polysaccharides improve cognitive function in D-galactose-induced aging mice by regulating the microbiota-gut-brain axis. J. Funct. Foods.

[97] Li H., Huang Y., Liang L., Li H., Li S., Feng Y., Feng S., Wu K., Wu F. (2024). The relationship between the gut microbiota and oxidative stress in the cognitive function of schizophrenia: A pilot study in China. Schizophr. Res..

[98] Liu Z., Wu C., Sun Z., Lin Z., Sun Y., Amjad N., Majid M., Basnet R., Li Z. (2025). Gut microbiota remodeling exacerbates neuroinflammation and cognitive dysfunction via the microbiota-gut-brain axis in prenatal VPA-exposed C57BL/6 mice offspring. Front. Immunol..

[99] Hyży A., Rozenek H., Gondek E., Jaworski M. (2025). Effect of antioxidants on the gut microbiome profile and brain functions: A review of randomized controlled trial studies. Foods.

[100] Faccinetto-Beltrán P., Reza-Zaldivar E.E., Curiel-Pedraza D.A., Canales-Aguirre A.A., Jacobo-Velázquez D.A. (2024). Docosahexaenoic acid (DHA), vitamin D3, and probiotics supplementation improve memory, glial reactivity, and oxidative stress biomarkers in an aluminum-induced cognitive impairment rat model. ACS Omega.

[101] Wang Z., Ma X., Shi W., Zhu W., Feng X., Xin H., Zhang Y., Cong B., Li Y. (2025). The Gut Microbiota Metabolite Butyrate Modulates Acute Stress-Induced Ferroptosis in the Prefrontal Cortex via the Gut–Brain Axis. Int. J. Mol. Sci..

[102] Erny D., Dokalis N., Mezö C., Castoldi A., Mossad O., Staszewski O., Frosch M., Villa M., Fuchs V., Mayer A. (2021). Microbiota-derived acetate enables the metabolic fitness of the brain innate immune system during health and disease. Cell Metab..

[103] Tu J., Zhang J., Chen G. (2025). Higher dietary butyrate intake is associated with better cognitive function in older adults: Evidence from a cross-sectional study. Front. Aging Neurosci..

[104] Li Y., Liu A., Chen K., Li L., Zhang X., Zou F., Zhang X., Meng X. (2024). Sodium butyrate alleviates lead-induced neuroinflammation and improves cognitive and memory impairment through the ACSS2/H3K9ac/BDNF pathway. Environ. Int..

[105] Cavaleri F., Bashar E. (2018). Potential synergies of β-hydroxybutyrate and butyrate on the modulation of metabolism, inflammation, cognition, and general health. J. Nutr. Metab..

[106] Yuan B., Liu M., Gong Y., Wang Z., Jin X., Xie G., Zhu M., Zhang X., Luo S., Qu Q. (2022). Sodium butyrate exerts antioxidant stress effects and attenuates Aβ25-35-induced cytotoxicity in PC12 cells. Arch. Biochem. Biophys..

[107] Xie A., Ensink E., Li P., Gordevičius J., Marshall L.L., George S., Pospisilik J.A., Aho V.T., Houser M.C., Pereira P.A. (2022). Bacterial butyrate in Parkinson’s disease is linked to epigenetic changes and depressive symptoms. Mov. Disord..

[108] Majumdar A., Venkatesh I.P.S., Swarup V., Basu A. (2024). Short-chain fatty acids abrogate Japanese encephalitis virus-induced inflammation in microglial cells via miR-200a-3p/ZBTB20/IKβα axis. Mbio.

[109] Rode J., Yang L., König J., Hutchinson A.N., Wall R., Venizelos N., Brummer R.-J., Rangel I., Vumma R. (2021). Butyrate rescues oxidative stress-induced transport deficits of tryptophan: Potential implication in affective or gut-brain axis disorders. Neuropsychobiology.

[110] Rose S., Bennuri S.C., Davis J.E., Wynne R., Slattery J.C., Tippett M., Delhey L., Melnyk S., Kahler S.G., MacFabe D.F. (2018). Butyrate enhances mitochondrial function during oxidative stress in cell lines from boys with autism. Transl. Psychiatry.

[111] Kim S.Y., Chae C.W., Lee H.J., Jung Y.H., Choi G.E., Kim J.S., Lim J.R., Lee J.E., Cho J.H., Park H. (2020). Sodium butyrate inhibits high cholesterol-induced neuronal amyloidogenesis by modulating NRF2 stabilization-mediated ROS levels: Involvement of NOX2 and SOD1. Cell Death Dis..

[112] Wang C., Zheng D., Weng F., Jin Y., He L. (2022). Sodium butyrate ameliorates the cognitive impairment of Alzheimer’s disease by regulating the metabolism of astrocytes. Psychopharmacology.

[113] Lu L.-L., Liu L.-Z., Li L., Hu Y.-Y., Xian X.-H., Li W.-B. (2024). Sodium butyrate improves cognitive dysfunction in high-fat diet/streptozotocin-induced type 2 diabetic mice by ameliorating hippocampal mitochondrial damage through regulating AMPK/PGC-1α pathway. Neuropharmacology.

[114] He Q., Ji L., Wang Y., Zhang Y., Wang H., Wang J., Zhu Q., Xie M., Ou W., Liu J. (2024). Acetate enables metabolic fitness and cognitive performance during sleep disruption. Cell Metab..

[115] Hu S., Kuwabara R., de Haan B.J., Smink A.M., de Vos P. (2020). Acetate and butyrate improve β-cell metabolism and mitochondrial respiration under oxidative stress. Int. J. Mol. Sci..

[116] Wen C., Xie T., Pan K., Deng Y., Zhao Z., Li N., Bian J., Deng X., Zha Y. (2020). Acetate attenuates perioperative neurocognitive disorders in aged mice. Aging.

[117] Osman A., Mervosh N.L., Strat A.N., Euston T.J., Zipursky G., Pollak R.M., Meckel K.R., Tyler S.R., Chan K.L., Grice A.B. (2023). Acetate supplementation rescues social deficits and alters transcriptional regulation in prefrontal cortex of Shank3 deficient mice. Brain Behav. Immun..

[118] Tanelian A., Nankova B., Hu F., Sahawneh J.D., Sabban E.L. (2023). Effect of acetate supplementation on traumatic stress-induced behavioral impairments in male rats. Neurobiol. Stress.

[119] Mazziotta C., Tognon M., Martini F., Torreggiani E., Rotondo J.C. (2023). Probiotics mechanism of action on immune cells and beneficial effects on human health. Cells.

[120] Maftei N.-M., Raileanu C.R., Balta A.A., Ambrose L., Boev M., Marin D.B., Lisa E.L. (2024). The potential impact of probiotics on human health: An update on their health-promoting properties. Microorganisms.

[121] Liu J., Sun J., Wang F., Yu X., Ling Z., Li H., Zhang H., Jin J., Chen W., Pang M. (2015). Neuroprotective effects of Clostridium butyricum against vascular dementia in mice via metabolic butyrate. BioMed Res. Int..

[122] Sarkar S.R., Mazumder P.M., Chatterjee K., Sarkar A., Adhikary M., Mukhopadhyay K., Banerjee S. (2021). Saccharomyces boulardii ameliorates gut dysbiosis associated cognitive decline. Physiol. Behav..

[123] Wang H., He S., Xin J., Zhang T., Sun N., Li L., Ni X., Zeng D., Ma H., Bai Y. (2021). Psychoactive effects of *Lactobacillus johnsonii* against restraint stress-induced memory dysfunction in mice through modulating intestinal inflammation and permeability—A study based on the gut–brain axis hypothesis. Front. Pharmacol..

[124] Detman A., Mielecki D., Chojnacka A., Salamon A., Błaszczyk M.K., Sikora A. (2019). Cell factories converting lactate and acetate to butyrate: *Clostridium butyricum* and microbial communities from dark fermentation bioreactors. Microb. Cell Factories.

[125] Cheng L.-H., Chou P.-Y., Hou A.-T., Huang C.-L., Shiu W.-L., Wang S. (2022). *Lactobacillus paracasei* PS23 improves cognitive deficits via modulating the hippocampal gene expression and the gut microbiota in D-galactose-induced aging mice. Food Funct..

[126] Wu Y., Niu X., Li P., Tong T., Wang Q., Zhang M., Li Y., Liu J., Li Z. (2023). Lactobacillaceae improve cognitive dysfunction via regulating gut microbiota and suppressing Aβ deposits and neuroinflammation in APP/PS1 mice. Arch. Microbiol..

[127] Wang Y., Wang B., Zeng Z., Liu R., Tang L., Meng X., Li W. (2023). *Bacillus amyloliquefaciens* SC06 attenuated high-fat diet induced anxiety-like behavior and social withdrawal of male mice by improving antioxidant capacity, intestinal barrier function and modulating intestinal dysbiosis. Behav. Brain Res..

[128] Wang X., Hu R., Lin F., Yang T., Lu Y., Sun Z., Li T., Chen J. (2024). *Lactobacillus reuteri* or *Lactobacillus rhamnosus* GG intervention facilitates gut barrier function, decreases corticosterone and ameliorates social behavior in LPS-exposed offspring. Food Res. Int..

[129] Chen H., Ouyang W., Cui X., Ma X., Hu S., Qing W., Tong J. (2025). miR-124 mediates the effects of gut microbial dysbiosis on brain function in chronic stressed mice. Behav. Brain Res..

[130] Mao L., Zeng Q., Su W., Song M., Li J., Xie M. (2021). Elevation of miR-146a inhibits BTG2/BAX expression to ameliorate postoperative cognitive dysfunction following probiotics (VSL# 3) treatment. Mol. Neurobiol..

[131] Akbari E., Asemi Z., Daneshvar Kakhaki R., Bahmani F., Kouchaki E., Tamtaji O.R., Hamidi G.A., Salami M. (2016). Effect of probiotic supplementation on cognitive function and metabolic status in Alzheimer’s disease: A randomized, double-blind and controlled trial. Front. Aging Neurosci..

[132] Tamtaji O.R., Heidari-Soureshjani R., Mirhosseini N., Kouchaki E., Bahmani F., Aghadavod E., Tajabadi-Ebrahimi M., Asemi Z. (2019). Probiotic and selenium co-supplementation, and the effects on clinical, metabolic and genetic status in Alzheimer’s disease: A randomized, double-blind, controlled trial. Clin. Nutr..

[133] Hsu Y.-C., Huang Y.-Y., Tsai S.-Y., Kuo Y.-W., Lin J.-H., Ho H.-H., Chen J.-F., Hsia K.-C., Sun Y. (2023). Efficacy of probiotic supplements on brain-derived neurotrophic factor, inflammatory biomarkers, oxidative stress and cognitive function in patients with Alzheimer’s dementia: A 12-week randomized, double-blind active-controlled study. Nutrients.

[134] Tkach S., Dorofeyev A., Kuzenko I., Boyko N., Falalyeyeva T., Boccuto L., Scarpellini E., Kobyliak N., Abenavoli L. (2022). Current status and future therapeutic options for fecal microbiota transplantation. Medicina.

[135] Zikou E., Koliaki C., Makrilakis K. (2024). The role of fecal microbiota transplantation (FMT) in the management of metabolic diseases in humans: A narrative review. Biomedicines.

[136] Fanizzi F., D’Amico F., Bombassaro I.Z., Zilli A., Furfaro F., Parigi T.L., Cicerone C., Fiorino G., Peyrin-Biroulet L., Danese S. (2024). The role of fecal microbiota transplantation in IBD. Microorganisms.

[137] Ren J., Wang Q., Hong H., Tang C. (2025). Fecal Microbiota Transplantation in Alzheimer’s Disease: Mechanistic Insights Through the Microbiota–Gut–Brain Axis and Therapeutic Prospects. Microorganisms.

[138] Zheng L., Jiao Y., Zhong H., Tan Y., Yin Y., Liu Y., Liu D., Wu M., Wang G., Huang J. (2024). Human-derived fecal microbiota transplantation alleviates social deficits of the BTBR mouse model of autism through a potential mechanism involving vitamin B6 metabolism. Msystems.

[139] Nirmalkar K., Qureshi F., Kang D.-W., Hahn J., Adams J.B., Krajmalnik-Brown R. (2022). Shotgun metagenomics study suggests alteration in sulfur metabolism and oxidative stress in children with autism and improvement after microbiota transfer therapy. Int. J. Mol. Sci..

[140] Abuaish S., Al-Otaibi N.M., Aabed K., Abujamel T.S., Alzahrani S.A., Alotaibi S.M., Bhat R.S., Arzoo S., Algahtani N., Moubayed N.M. (2022). The efficacy of fecal transplantation and bifidobacterium supplementation in ameliorating propionic acid-induced behavioral and biochemical autistic features in juvenile male rats. J. Mol. Neurosci..

[141] Duan R., Hou J., Wang X., Huang Z., Cao H., Hu J., Peng Q., Duan H., Wang Q., Chen X. (2023). *Prevotella histicola* transplantation ameliorates cognitive impairment and decreases oxidative stress in vascular dementia rats. Brain Sci..

[142] Sionek B., Szydłowska A. (2025). Probiotics and Prebiotics in the Aspect of Health Benefits and the Development of Novel Plant-Based Functional Food. Appl. Sci..

[143] Gao X., Hu S., Liu Y., De Alwis S.S.S., Yu Y., Li Z., Wang Z., Liu J. (2025). Dietary Fiber as Prebiotics: A Mitigation Strategy for Metabolic Diseases. Foods.

[144] Ghodbane I., Boukhechem S., Bougherara H., Monnoye M., Oubira I., Lakhdara N., Gerard P., Dib A.L. (2025). The Role of Plant-Derived Prebiotics in Obesity Management: Mechanisms, Efficacy, and Active Compounds. Biol. Life Sci. Forum.

[145] Li M., Zhang C., Xiao X., Zhu M., Quan W., Liu X., Zhang S., Liu Z. (2023). Theaflavins in black tea mitigate aging-associated cognitive dysfunction via the microbiota–gut–brain axis. J. Agric. Food Chem..

[146] Fekete M., Lehoczki A., Major D., Fazekas-Pongor V., Csípő T., Tarantini S., Csizmadia Z., Varga J.T. (2024). Exploring the influence of gut–brain axis modulation on cognitive health: A comprehensive review of prebiotics, probiotics, and symbiotics. Nutrients.

[147] Bevilacqua A., Campaniello D., Speranza B., Racioppo A., Sinigaglia M., Corbo M.R. (2024). An update on prebiotics and on their health effects. Foods.

[148] Liu B., Chen B., Yi J., Long H., Wen H., Tian F., Liu Y., Xiao L., Li L. (2022). Liuwei dihuang decoction alleviates cognitive dysfunction in mice with D-galactose-induced aging by regulating lipid metabolism and oxidative stress via the microbiota-gut-brain axis. Front. Neurosci..

[149] Liu Q., Xi Y., Wang Q., Liu J., Li P., Meng X., Liu K., Chen W., Liu X., Liu Z. (2021). Mannan oligosaccharide attenuates cognitive and behavioral disorders in the 5xFAD Alzheimer’s disease mouse model via regulating the gut microbiota-brain axis. Brain Behav. Immun..

[150] Gao Y., Li B., Liu H., Tian Y., Gu C., Du X., Bu R., Gao J., Liu Y., Li G. (2021). Cistanche deserticola polysaccharides alleviate cognitive decline in aging model mice by restoring the gut microbiota-brain axis. Aging.

[151] Chen S.-Y., Weng M.-H., Li Z.-Y., Wang G.-Y., Yen G.-C. (2022). Protective effects of camellia and olive oils against cognitive impairment via gut microbiota-brain communication in rats. Food Funct..

[152] Yang L., Wang Y., Zheng G., Li Z., Mei J. (2023). Resveratrol-loaded selenium/chitosan nano-flowers alleviate glucolipid metabolism disorder-associated cognitive impairment in Alzheimer’s disease. Int. J. Biol. Macromol..

[153] Li M., Zhu M., Quan W., Huang W., Liu X., Zhang C., Lu B., Xiao X., Liu Z. (2023). Acteoside palliates d-galactose induced cognitive impairment by regulating intestinal homeostasis. Food Chem..

[154] Zhang N., Jing P. (2023). Red cabbage anthocyanins attenuate cognitive impairment by attenuating neuroinflammation and regulating gut microbiota in aging mice. J. Agric. Food Chem..

[155] Million M., Armstrong N., Khelaifia S., Guilhot E., Richez M., Lagier J.-C., Dubourg G., Chabriere E., Raoult D. (2020). The antioxidants glutathione, ascorbic acid and uric acid maintain butyrate production by human gut clostridia in the presence of oxygen in vitro. Sci. Rep..

[156] Gao Y., Xu Y., Yin J. (2022). Selenomethionine ameliorates cognitive impairment, decreases hippocampal oxidative stress and attenuates dysbiosis in D-galactose-treated mice. Antioxidants.

[157] Lin X., Bo H., Gu J., Yi X., Zhang P., Liu R., Li H., Sun G., Lin C.-H. (2022). Astaxanthin, a carotenoid antioxidant, pretreatment alleviates cognitive deficits in aircraft noised mice by attenuating inflammatory and oxidative damage to the gut, heart and hippocampus. Biomed. Pharmacother..

[158] Dong W., Peng Y., Chen G., Xie Z., Xu W., Zhou W., Mi J., Lu L., Sun Y., Zeng X. (2024). 2-O-β-D-Glucopyranosyl-L-ascorbic acid, an ascorbic acid derivative isolated from the fruits of *Lycium barbarum* L., ameliorates high fructose-induced neuroinflammation in mice: Involvement of gut microbiota and leaky gut. Food Sci. Hum. Wellness.

[159] Li S., Yang P., Cai X., He M., He Y., He F. (2025). Vitamin C supplementation mitigates mild cognitive impairment in mice subjected to D-galactose: Insights into intestinal flora and derived SCFAs. Eur. J. Pharmacol..

[160] Chatterjee K., Mazumder P.M., Sarkar S.R., Saha R., Chatterjee A., Sarkar B., Banerjee S. (2023). Neuroprotective effect of Vitamin K2 against gut dysbiosis associated cognitive decline. Physiol. Behav..

[161] Dou X., Cai S., Liu Y., Wang J., Li H., Gao D. (2025). Synergistic Effect Evaluation and Mechanism Investigation of Vitamin B6 and B12 in Models of Neuroinflammation. Int. J. Mol. Sci..

[162] Zwierz M., Suprunowicz M., Mrozek K., Pietruszkiewicz J., Oracz A.J., Konarzewska B., Waszkiewicz N. (2025). Vitamin B12 and autism spectrum disorder: A review of current evidence. Nutrients.

[163] Theodosis-Nobelos P., Rekka E.A. (2024). The antioxidant potential of vitamins and their implication in metabolic abnormalities. Nutrients.

[164] Obeid R., Shannan B., Herrmann W. (2011). Advanced glycation end products overload might explain intracellular cobalamin deficiency in renal dysfunction, diabetes and aging. Med. Hypotheses.

[165] Park S., Kang S., Kim D.S. (2019). Folate and vitamin B-12 deficiencies additively impaire memory function and disturb the gut microbiota in amyloid-β infused rats. Int. J. Vitam. Nutr. Res..

[166] Robea M.A., Ilie O.D., Nicoara M.N., Solcan G., Romila L.E., Ureche D., Ciobica A. (2024). Vitamin B12 Ameliorates Pesticide-Induced Sociability Impairment in Zebrafish (*Danio rerio*): A Prospective Controlled Intervention Study. Animals.

[167] Liu F., Liu Y., Feng Y., Zhao J., Wang M., Ye M., Zhang Y., Gan X., Pan Q., Shen J. (2025). Folate mediates cognitive impairment of aged people with periodontitis. Nutr. Neurosci..

[168] Li B., Wu K., Duan G., Yin W., Lei M., Yan Y., Ren Y., Zhang C. (2024). Folic acid and taurine alleviate the impairment of redox status, immunity, rumen microbial composition and fermentation of lambs under heat stress. Animals.

[169] Darbandi Z.K., Amirahmadi S., Goudarzi I., Hosseini M., Rajabian A. (2024). Folic acid improved memory and learning function in a rat model of neuroinflammation induced by lipopolysaccharide. Inflammopharmacology.

[170] Novochadlo M., Goldim M.P., Bonfante S., Joaquim L., Mathias K., Metzker K., Machado R.S., Lanzzarin E., Bernades G., Bagio E. (2021). Folic acid alleviates the blood brain barrier permeability and oxidative stress and prevents cognitive decline in sepsis-surviving rats. Microvasc. Res..

[171] Wan Z., Zheng J., Zhu Z., Sang L., Zhu J., Luo S., Zhao Y., Wang R., Zhang Y., Hao K. (2022). Intermediate role of gut microbiota in vitamin B nutrition and its influences on human health. Front. Nutr..

[172] Nakano E., Higgins J.A., Powers H.J. (2001). Folate protects against oxidative modification of human LDL. Br. J. Nutr..

[173] Joshi R., Adhikari S., Patro B., Chattopadhyay S., Mukherjee T. (2001). Free radical scavenging behavior of folic acid: Evidence for possible antioxidant activity. Free Radic. Biol. Med..

[174] Jang J., Kim S.R., Lee J.E., Lee S., Son H.J., Choe W., Yoon K.-S., Kim S.S., Yeo E.-J., Kang I. (2023). Molecular mechanisms of neuroprotection by ketone bodies and ketogenic diet in cerebral ischemia and neurodegenerative diseases. Int. J. Mol. Sci..

[175] Rubio C., López-Landa A., Romo-Parra H., Rubio-Osornio M. (2025). Impact of the ketogenic diet on neurological diseases: A review. Life.

[176] Greco T., Glenn T.C., Hovda D.A., Prins M.L. (2016). Ketogenic diet decreases oxidative stress and improves mitochondrial respiratory complex activity. J. Cereb. Blood Flow Metab..

[177] Jiang J., Pan H., Shen F., Tan Y., Chen S. (2023). Ketogenic diet alleviates cognitive dysfunction and neuroinflammation in APP/PS1 mice via the Nrf2/HO-1 and NF-κB signaling pathways. Neural Regen. Res..

[178] Qin Y., Bai D., Tang M., Zhang M., Zhao L., Li J., Yang R., Jiang G. (2023). Ketogenic diet alleviates brain iron deposition and cognitive dysfunction via Nrf2-mediated ferroptosis pathway in APP/PS1 mouse. Brain Res..

[179] Qiao Q., Tian S., Zhang Y., Che L., Li Q., Qu Z., Wang W. (2024). A ketogenic diet may improve cognitive function in rats with temporal lobe epilepsy by regulating endoplasmic reticulum stress and synaptic plasticity. Mol. Neurobiol..

[180] Li C., Ma Y., Chai X., Feng X., Feng W., Zhao Y., Cui C., Wang J., Zhao S., Zhu X. (2024). Ketogenic diet attenuates cognitive dysfunctions induced by hypoglycemia via inhibiting endoplasmic reticulum stress-dependent pathways. Food Funct..

[181] Abdel-Aziz R.H., Ahmed O.G., Mahmoud A.M., Abd-Elhafeez H.H., Abd-Elsamiee L., Toghan R. (2025). Effect of Ketogenic Diet on Cognitive Dysfunction associated with Alzheimer’s disease. SVU-Int. J. Med. Sci..

[182] Olivito I., Avolio E., Minervini D., Soda T., Rocca C., Angelone T., Iaquinta F.S., Bellizzi D., De Rango F., Bruno R. (2023). Ketogenic diet ameliorates autism spectrum disorders-like behaviors via reduced inflammatory factors and microbiota remodeling in BTBR T+ Itpr3tf/J mice. Exp. Neurol..

[183] Allan N.P., Yamamoto B.Y., Kunihiro B.P., Nunokawa C.K., Rubas N.C., Wells R.K., Umeda L., Phankitnirundorn K., Torres A., Peres R. (2024). Ketogenic diet induced shifts in the gut microbiome associate with changes to inflammatory cytokines and brain-related miRNAs in children with autism Spectrum disorder. Nutrients.

[184] Lu M., Yang Y., Xu Y., Wang X., Li B., Le G., Xie Y. (2023). Dietary methionine restriction alleviates choline-induced tri-methylamine-N-oxide (TMAO) elevation by manipulating gut microbiota in mice. Nutrients.

[185] Yang M., Xie Q., Xiao Y., Xia M., Chen J., Tan B.-E., Yin Y. (2024). Dietary methionine restriction improves gut health and alters the plasma metabolomic profile in rats by modulating the composition of the gut microbiota. Int. J. Mol. Sci..

[186] Wallis K.F., Melnyk S.B., Miousse I.R. (2020). Sex-specific effects of dietary methionine restriction on the intestinal microbiome. Nutrients.

[187] Yang Y., Zhang Y., Xu Y., Luo T., Ge Y., Jiang Y., Shi Y., Sun J., Le G. (2019). Dietary methionine restriction improves the gut microbiota and reduces intestinal permeability and inflammation in high-fat-fed mice. Food Funct..

[188] Wu G., Shi Y., Han L., Feng C., Ge Y., Yu Y., Tang X., Cheng X., Sun J., Le G.-W. (2020). Dietary methionine restriction ameliorated fat accumulation, systemic inflammation, and increased energy metabolism by altering gut microbiota in middle-aged mice administered different fat diets. J. Agric. Food Chem..

[189] Xu Y., Yang Y., Li B., Xie Y., Shi Y., Le G. (2022). Dietary methionine restriction improves gut microbiota composition and prevents cognitive impairment in D-galactose-induced aging mice. Food Funct..

